# mRNA vaccine-induced SARS-CoV-2 spike-specific IFN-γ and IL-2 T-cell responses are predictive of serological neutralization and are transiently enhanced by pre-existing cross-reactive immunity

**DOI:** 10.1128/jvi.01685-24

**Published:** 2025-01-31

**Authors:** Philip Samaan, Chapin S. Korosec, Patrick Budylowski, Serena L. L. Chau, Adrian Pasculescu, Freda Qi, Melanie Delgado-Brand, Tulunay R. Tursun, Geneviève Mailhot, Roya Monica Dayam, Corey R. Arnold, Marc-André Langlois, Justin Mendoza, Thomas Morningstar, Ryan Law, Erik Mihelic, Salma Sheikh-Mohamed, Eric Yixiao Cao, Nimitha Paul, Anjali Patel, Keelia Quinn de Launay, Jamie M. Boyd, Alyson Takaoka, Karen Colwill, Vitaliy Matveev, Feng Yun Yue, Allison McGeer, Sharon Straus, Anne-Claude Gingras, Jane M. Heffernen, Mario Ostrowski

**Affiliations:** 1 Department of Laboratory Medicine and Pathobiology, University of Toronto 7938 , Toronto, Ontario, Canada; 2 Modelling Infection and Immunity Lab, Mathematics and Statistics, York University 375295 , Toronto, Ontario, Canada; 3 Center for Disease Modelling, Mathematics and Statistics, York University 375295 , Toronto, Ontario, Canada; 4 Institute of Medical Science, University of Toronto 686133 , Toronto, Ontario, Canada; 5 Department of Medicine, University of Toronto 233846 , Toronto, Ontario, Canada; 6 Lunenfeld-Tanenbaum Research Institute, Sinai Health 90755 , Toronto, Ontario, Canada; 7 Department of Biochemistry, Microbiology and Immunology, University of Ottawa 151173 , Ottawa, Ontario, Canada; 8 Department of Immunology, University of Toronto 7938 , Toronto, Ontario, Canada; 9 Unity Health Toronto, St Michael's Hospital 10071 , Toronto, Ontario, Canada; 10 Department of Molecular Genetics, University of Toronto 204248 , Toronto, Ontario, Canada; 11 Keenan Research Center for Biomedical Science, St Michael's Hospital Keenan 568429 , Toronto, Ontario, Canada; Loyola University Chicago - Health Sciences Campus, Maywood, Illinois, USA

**Keywords:** SARS-CoV-2, T-cell immunity, humoral immunity, mRNA vaccines, cross-reactivity, hybrid immunity

## Abstract

The contributions of severe acute respiratory syndrome coronavirus 2 (SARS-CoV-2)-specific T cells to vaccine efficacy and durability are unclear. We investigated relationships between mRNA vaccine-induced spike-specific interferon- gamma (IFN-γ) and interleukin-2 (IL-2) T-cell responses and neutralizing antibody development in long-term care home staff doubly vaccinated with BNT162b2 or mRNA-1273. The impacts of pre-existing cross-reactive T-cell immunity on cellular and humoral responses to vaccination were additionally assessed. Mathematical modeling of the kinetics of spike-specific IFN-γ and IL-2 T-cell responses over 6 months post-second dose was bifurcated into recipients who exhibited gradual increases with doubling times of 155 and 167 days or decreases with half-lives of 165 and 132 days, respectively. Differences in kinetics did not correlate with clinical phenotypes. Serological anti-spike IgG, anti-receptor binding domain (RBD) IgG, anti-spike IgA, and anti-RBD IgA antibody levels otherwise decayed in all participants with half-lives of 63, 57, 79, and 46 days, respectively, alongside waning neutralizing capacity (*t*_1/2_ = 408 days). Spike-specific T-cell responses induced at 2–6 weeks positively correlated with live viral neutralization at 6 months post-second dose, especially in hybrid immune individuals. Participants with pre-existing cross-reactive T-cell immunity to SARS-CoV-2 exhibited greater spike-specific T-cell responses, reduced anti-RBD IgA antibody levels, and a trending increase in neutralization at 2–6 weeks post-second dose. Non-spike-specific T-cells predominantly targeted SARS-CoV-2 non-structural protein at 6 months post-second dose in cross-reactive participants. mRNA vaccination was lastly shown to induce off-target T-cell responses against unrelated antigens. In summary, vaccine-induced spike-specific T-cell immunity appeared to influence serological neutralizing capacity, with only a modest effect induced by pre-existing cross-reactivity.

**IMPORTANCE:**

Our findings provide valuable insights into the potential contributions of mRNA vaccine-induced spike-specific T-cell responses to the durability of neutralizing antibody levels in both uninfected and hybrid immune recipients. Our study additionally sheds light on the precise impacts of pre-existing cross-reactive T-cell immunity to severe acute respiratory syndrome coronavirus 2 on the magnitude and kinetics of cellular and humoral responses to vaccination. Accordingly, our data will help optimize the development of next-generation T cell-based coronavirus vaccines and vaccine regimens to maximize efficacy and durability.

## INTRODUCTION

The coronavirus disease 2019 (COVID-19) pandemic, caused by the severe acute respiratory syndrome coronavirus 2 (SARS-CoV-2) virus, continues to pose a serious threat to public health since its emergence in Wuhan, China, in December 2019. Immunological correlates of protection against infection, symptomatic disease, and death are still unclear (1). Investigating these correlates can aid in the identification of surrogate markers that can be used to predict SARS-CoV-2 vaccine efficacy and durability on an individual basis.

T cells are key players in the adaptive immune response to invading pathogens (2). In COVID-19, the earlier induction of robust SARS-CoV-2-specific T-cell responses during acute infection are well correlated with viral control and milder disease course (3–6). The importance of T cells in viral clearance is likewise highlighted within the context of vaccination. As SARS-CoV-2 rapidly evolved to evade neutralizing humoral responses over time, vaccine regimens were rendered progressively less efficacious against symptomatic infection (7–9). Yet, the same vaccine regimens maintained a high degree of protection against severe disease (7–9), which aligned with accompanying reports indicating that vaccine-induced spike-specific T-cell responses across SARS-CoV-2 variants of concern (VOCs) are mostly preserved (10, 11). Accordingly, much interest has been garnered in the role of T cells in vaccination against SARS-CoV-2.

CD4^+^ T cells are critical for directing the adaptive immune response to viral pathogens. Firstly, CD4^+^ T cells can provide indirect help to CD8^+^ T cells by licensing antigen-presenting dendritic cells, which in turn become more efficient at activating CD8^+^ T cells (12). Activated CD8^+^ T cells sequentially proliferate and differentiate into cytotoxic T lymphocytes that kill virally infected target cells (12). A significant negative moderate correlation between SARS-CoV-2-specific CD8^+^ T-cell responses and peak disease severity in COVID-19 patients has notably been observed in a previous report (5). In this regard, memory CD4^+^ T cells may be crucial for the preservation of protection against severe disease conferred by vaccine regimens across VOCs (9, 13, 14). Secondly, CD4^+^ T cells provide essential help to B cells for the development of neutralizing antibodies (15, 16). Previous studies have reported that the potency and earlier induction of SARS-CoV-2-specific neutralizing antibodies are predictive of milder disease course and survival in COVID-19 patients (17, 18). Accordingly, the conservation of SARS-CoV-2-specific T-cell responses across VOCs may have preserved the ability to quickly induce robust neutralizing humoral responses against infection (19), preventing severe disease.

Within the context of natural infection, several previous studies have already shown significant positive associations between SARS-CoV-2-specific CD4^+^ T-cell responses and serological antibody levels and neutralization (4, 5, 20–22). However, these associations have been evaluated in very few studies to date within the context of vaccination (23, 24). Most importantly, the precise relationships between the kinetics of vaccine-induced spike-specific CD4^+^ T-cell responses and neutralizing antibody levels have not been thoroughly investigated.

It was previously found that the neutralizing activity of acute and convalescent plasma/serum is mostly facilitated by spike- and receptor binding domain (RBD)-specific IgM and IgG1 antibodies (25). Serological IgA antibodies were also reported to contribute to neutralizing activity (25) and have been observed to play a potential role in protection against infection (26). Considering the collective contributions of IFN-γ and IL-2 secreted by CD4^+^ T helper cells to B-cell proliferation, differentiation, and survival along with antibody class switching to IgG and IgA (20, 27–32), we hypothesize that spike-specific IFN-γ and IL-2 T-cell responses to vaccination will enhance neutralizing antibody levels against SARS-CoV-2.

Previous exposure to human common-cold coronaviruses (HCoVs) may potentially impact both spike-specific CD4^+^ T-cell and neutralizing humoral responses to vaccination due to their significant homologies with SARS-CoV-2 (33). Multiple studies have shown that at the beginning of the COVID-19 pandemic, 20%–50% of healthy, unexposed individuals exhibited pre-existing cross-reactive CD4^+^ T-cell responses to SARS-CoV-2 that were attributable to previous HCoV infections (34–37). The impact of this phenomenon on immune responses to vaccination against SARS-CoV-2 has to date only been investigated in few studies (33). Previous reports collectively suggest that pre-existing cross-reactive cellular immunity may boost mRNA vaccine-induced spike-specific CD4^+^ T-cell responses and neutralizing antibody levels against SARS-CoV-2 (33, 38–40). However, precise impacts on the kinetics of these responses are yet unclear.

It is well established in literature that memory T cells generally produce more robust and rapid responses to secondary infection compared to naïve T cells (41). Additionally, memory CD4^+^ T cells provide accelerated help and promote significantly earlier class switching in primary B cells compared to naïve CD4^+^ T cells over the course of an infection (19). Accordingly, we hypothesize that individuals with pre-existing cross-reactive T-cell immunity to SARS-CoV-2 will exhibit boosts in vaccine-induced spike-specific T-cell responses and neutralizing antibody levels.

Investigating the precise relationships between the kinetics of vaccine-induced cellular and humoral responses to SARS-CoV-2 and the impact of pre-existing cross-reactive T-cell immunity is of paramount importance for optimizing the development of next-generation T cell-based coronavirus vaccines and vaccine regimens to maximize efficacy and durability. To pursue our objectives, dual-color ELISpot, enzyme-linked immunosorbent assay (ELISA), and live SARS-CoV-2 neutralization assays were deployed to longitudinally assess SARS-CoV-2 spike-specific IFN-γ and IL-2 T-cell responses, serological anti-spike/RBD IgG and IgA antibody levels, and neutralizing capacity in high-risk long-term care home (LTCH) staff up to 6 months post-second dose of BNT162b2 or mRNA-1273. Non-linear mixed-effects mathematical modeling was additionally deployed to precisely evaluate the kinetics of cellular and humoral responses to mRNA vaccination overtime.

## MATERIALS AND METHODS

### Study cohort

From February to December 2021, peripheral blood mononuclear cell (PBMC) and serum samples were collected from 139 LTCH staff recruited from 12 LTCHs across the Greater Toronto Area and St. Champlain regions of Ontario, Canada. Samples were collected up to 6 months post-second dose of BNT162b2 or mRNA-1253. The LTCH staff recruited into the study were particularly those who worked in high-risk long-term care homes and resided in higher-density households within high-risk neighborhoods. LTCH staff in these regions were heavily impacted by the first and second waves of the COVID-19 pandemic (42). Vaccine uptake was later associated with reduced incidences of COVID-19 (42).

### COVID-19 screening of LTCH staff

In accordance with the directive issued by the Minister of Long-Term Care of Ontario regarding LTCH SARS-CoV-2 surveillance testing and access to LTCHs, the cohort studied in this paper was required to take one of the following:

- One PCR test and one rapid antigen test (RAT) on separate days within a period of 7 days (the period between PCR testing and RAT was required to be as close to 7 days as practically achievable);
- A RAT at least twice a week on separate days if fully vaccinated (at least 2 doses) against COVID-19; or
- A RAT at least three times per week on separate days if not fully vaccinated against COVID-19. COVID-19 screening of LTCH staff was continuously conducted across all timepoints in this study.

### PBMC isolation

PBMCs were isolated from whole blood samples collected in 8.5 mL BD Vacutainer glass collection tubes with acid citrate dextrose used as an anticoagulant (Fisher Scientific). Two tubes were collected from each participant. Whole blood was layered on Ficoll-Paque Plus (GE Healthcare) in 50 mL SepMate tubes (Stemcell Technologies) and centrifuged at 1,200 × *g* for 10 minutes. The buffy coat was then isolated and washed twice with 2% heat-inactivated fetal bovine serum (FBS; Wisent) in Dulbecco’s phosphate-buffered saline (D-PBS; Wisent) before being resuspended in R-10 medium. R-10 medium consisted of a mixture of RPMI 1640 (Wisent), 10% FBS (Wisent), 10 mM HEPES (Wisent), 2 mM L-glutamine (Wisent), and 100 UI/mL of penicillin-streptomycin (Wisent). Resuspended PBMCs were then diluted 1:1 with freezing medium consisting of 20% dimethyl sulfoxide (DMSO; Sigma-Aldrich) and 80% FBS and subsequently aliquoted for storage at −150°C.

### Serum isolation

Serum was isolated from whole blood collected in 3.5 mL BD Vacutainer serum separation tubes containing spray-coated silica and a polymer gel for serum separation (Fisher Scientific). One tube was collected from each participant. Tubes were centrifuged at 1,200 × *g* for 10 minutes before serum was subsequently isolated and aliquoted for storage at −80°C.

### SARS-CoV-2 peptide masterpool synthesis

SARS-CoV-2 15-mer peptides from nucleocapsid phosphoprotein (N), envelope glycoprotein (E), membrane glycoprotein (M), and non-structural protein (NSP) were synthesized by GeneScript (Piscataway, NJ) using the wild-type SARS-CoV-2 reference sequence (accession number NC_045512.2, NCBI). The SARS-CoV-2 spike glycoprotein masterpool was obtained as a PepMix (Swiss-Prot ID: P0DTC2) from JPT (Berlin, Germany). The N, E, M, and S masterpools were, respectively, consisted of 102, 12, 49, and 315 (split into two pools of 158 and 157) 15-mer peptides with 11 amino acid overlaps. Each of the four aforementioned masterpools spanned the entirety of their corresponding antigens. The NSP masterpool consisted of 26 15-mer peptides that were collectively derived from 14 non-structural antigens listed in Table S1. These peptides were previously reported to elicit cross-reactive responses toward SARS-CoV-2 in healthy, unexposed individuals (34). All peptides were reconstituted in 100% DMSO and aliquoted for storage at −80°C until needed.

### *Ex vivo* ELISpot assay

To perform *ex vivo* ELISpot assays, MSIPS4W plates (Millipore) were first activated with 35% ethanol. Activated plates were then washed with molecular grade sterile water (Wisent) before coating with 10 µg/mL of monoclonal IFN-γ (1-D1K) and 10 µg/mL of monoclonal IL-2 (MT2A91/2C95) capture antibodies (Mabtech). The coated plates were wrapped in parafilm wax and allowed to incubate overnight at 4°C. Following incubation, coated plates were washed with D-PBS and blocked with R-10 medium for 1 h at 37°C, 5% CO_2_. During this time, PBMCs were thawed and rested for at least 2 h at 37°C, 5% CO_2_. Following incubation, plates were washed with D-PBS and PBMCs were plated at 250,000 cells per well. Over a period of 24 h at 37°C, 5% CO_2_, PBMCs were then stimulated with N, E, M, S, or NSP masterpools at a concentration of 1 µg/mL. As positive controls to assess T-cell functionality, PBMCs were stimulated with cytomegalovirus, Epstein-Barr virus, influenza (CEF) (NIH HIV Reagent Program, #ARP-9808) and cytomegalovirus, Epstein-Barr virus, influenza, tetanus toxin, adenovirus 5 (CEFTA) (Mabtech, #3617-1) peptide pools at 1 µg/mL. As a positive control to assess the assay’s functionality, PBMCs were stimulated with staphylococcal enterotoxin B (SEB; Sigma-Aldrich, #S4881) at 0.1 µg/mL. At the end of the incubation period, PBMCs were discarded, and plates were washed with 0.05% Tween 20 (BioShop) in D-PBS. Plates were subsequently coated with a cocktail of alkaline phosphatase (ALP)-conjugated monoclonal IFN-γ detection antibody (7-B6-1; Mabtech) at 1:500 dilution and biotinylated monoclonal IL-2 detection antibody (MT8G10; Mabtech) at 0.25 µg/mL in D-PBS. Plates were allowed to incubate for 2 h at room temperature (RT) in the dark. After incubating, plates were washed again with 0.05% Tween 20 in D-PBS before streptavidin-horseradish peroxidase (HRP) conjugate was plated at 1:1,000 dilution in D-PBS. After incubating for another hour at RT in the dark, plates were washed with molecular-grade sterile water. Spots were lastly developed by first treating wells with Vector Blue developing reagent for ALP (Vector Laboratories) for 15 minutes at RT in the dark. Plates were subsequently washed with molecular-grade sterile water before wells were treated with Vector NovaRed developing reagent for HRP (Vector Laboratories) for 8 minutes at RT in the dark. Plates were then washed a final time with Milli-Q water and allowed to dry at 4°C in the dark. Spots in each well were quantified using an ImmunoSpot S3 Analyzer (Cellular Technology Limited).

To analyze the data, the average number of spots + 2 SD per million PBMC in negative control (DMSO) wells, or a minimum of 10 spots per million PBMC, whichever is greater, was subtracted from gross spot counts per million PBMC in treatment wells. Resulting net spot counts greater than 0 were considered positive.

### *In vitro* PBMC expansion

To expand PBMCs *in vitro*, cryopreserved PBMCs were thawed and washed twice with R-H10 medium consisting of a mixture of RPMI 1640 (Wisent), 10% heat-inactivated human AB serum (Wisent), 10 mM HEPES (Wisent), 2 mM GlutaMAX (Thermo Fisher Scientific), 100 UI/mL of penicillin-streptomycin (Wisent), 1 mM sodium pyruvate (Thermo Fisher Scientific), and 50 µM 2-mercaptoethanol (Thermo Fisher Scientific). PBMCs were adjusted to a final concentration of 2 × 10^6^ PBMC/mL before being plated at 400,000 cells (200 µL) per well per condition within a tissue culture-treated 96-well round-bottom polystyrene plate. After plating, PBMCs were treated with 0.1% DMSO (negative control) or with 0.1 µg/mL of NSP, CEF, CEFTA, or SEB, and incubated at 37°C, 5% CO_2_ for a total of 7 days. On day 1 of incubation (with cell plating being day 0), PBMCs treated with 0.1% DMSO or 0.1 µg/mL of NSP, CEF, or CEFTA were supplemented with 10 IU/mL of recombinant human IL-2 (rh IL-2; R&D Systems) and 10 ng/mL of recombinant human IL-7 (rh IL-7; R&D Systems). PBMCs treated with 0.1 µg/mL of SEB were supplemented with 10 ng/mL of rh IL-7 alone. By day 5 of incubation, half the culture medium in each well was carefully aspirated and replaced with fresh R-H10 medium. DMSO, NSP, CEF, and CEFTA wells were additionally re-supplemented with 10 IU/mL of rh IL-2 and 10 ng/mL of rh IL-7, while SEB wells were re-supplemented with 10 ng/mL of rh IL-7 alone. Lastly, on day 7, PBMCs were washed twice with R-10 medium after centrifuging at 300 × *g* for 5 minutes before being assayed by ELISpot.

### Chemiluminescent ELISA for detection of SARS-CoV-2-specific IgG and IgA antibodies in serum

A chemiluminescent ELISA was used to measure levels of serological IgG and IgA antibody to the full-length spike trimer, the RBD, and nucleocapsid of the ancestral SARS-CoV-2 virus, as previously described (43, 44). Briefly, LUMITRAC 600 high-binding white polystyrene 384-well microplates (Greiner Bio-One, #781074; VWR, #82051-268) were first pre-coated overnight with 10 µL per well of antigen (Ag): 50 ng of full-length spike trimer (SmT1), 20 ng of RBD (331-521), and 7 ng of nucleocapsid. All antigens were supplied by the National Research Council of Canada (NRC). Following overnight incubation, microplates were washed four times at RT with 100 µL per well of PBS-T before each of the following steps. Step 1: wells were blocked for 1 h in 80 µL of 5% Blocker BLOTTO (Thermo Fisher Scientific, #37530). Step 2: 10 µL of serum diluted to 1:160, 1:640, 1:2,560, or 1:40,960 with 1% Blocker BLOTTO in PBS-T was added to each well and incubated for 2 h. Step 3: 10 µL of HRP-conjugated human anti-IgG (IgG#5 by NRC, 0.9 ng/well) or HRP-conjugated human anti-IgA (Jackson ImmunoResearch, #109-035-127, 0.8 ng/well) diluted with 1% Blocker BLOTTO in PBS-T was added to each well, followed by a 1 h incubation. Step 4: 10 µL of ELISA Pico Chemiluminescent Substrate (Thermo Fisher Scientific, #37069, diluted 1:4 in Milli-Q distilled H_2_O) was added and incubated for 5–8 minutes. Chemiluminescence was read on an EnVision 2105 Multimode Plate Reader (Perkin Elmer) at 100 ms/well using an ultra-sensitive detector. Raw chemiluminescent values were normalized to a synthetic standard included on each assay plate (for IgG: VHH72-Fc supplied by NRC for spike/RBD or an anti-nucleocapsid IgG Ab from Genescript, #A02039; for IgA: anti-spike CR3022 from Absolute Antibody, #Ab01680-16.0, and anti-nucleocapsid CR3018 from Absolute Antibody, #Ab01690-16.0). Computed relative ratios were further converted to binding Ab units (BAU/mL) using the WHO International Standard 20/136 as the calibrant (43). A positivity threshold for the 1:160 dilution was established as being 3SD from the mean of negative control samples, as previously described (43).

All measures of serological antibody levels that did not meet positivity thresholds were plotted as 0.

### Chemiluminescent direct ELISAs for HCoV spike-specific IgG antibody detection in serum

Automated chemiluminescent ELISAs were based upon and optimized from assays first described in reference 45. Assays were performed using Hamilton Microlab STAR robotic liquid handlers at the University of Ottawa’s Serology and Diagnostics High-Throughput Facility (Faculty of Medicine). To prepare assay plates, antigens (spike trimers from seasonal coronavirus strains: 229E [DAGC134], HKU1 [DAGC132], OC43 [DAGC131], and NL63 [DAGC133]; Creative Diagnostics) were diluted in PBS and applied to wells of a 384-well high-binding polystyrene Nunc plate (Thermo Fisher Scientific, #460372) at a final amount of 50 ng/well. Antigen-coated plates were centrifuged briefly at approximately 2,000 × *g* in a plate spinner (Fisher Scientific) to ensure even coating then incubated on a rocker at 4°C (minimum 4 h, up to 3 days). On the day of the assay, antigen-coated plates were washed, and wells were blocked with 80 µL of 3% wt/vol skim milk powder dissolved in PBS + 0.1% Tween (PBS-T) for 1 h. Samples and controls were diluted at 1:100, 1:1,000, and 1:10,000 in 1% wt/vol skim milk powder in PBS-T in a 96-well deep-well plate. Following the blocking incubation, plates were washed, and 10 µL of diluted samples and controls were added to their respective wells. Plates were incubated for 2 h and wells were then washed. Anti-IgG (NRC anti-hIgG#5-HRP fusion) was diluted at 1:5,400 in 1% wt/vol skim milk powder in PBS-T, and 10 µL was added to each well and incubated for 1 h. Plates were washed and 10 µL of ELISA Pico Chemiluminescent Substrate (Thermo) diluted 1:2 in Milli-Q H_2_O was dispensed into each well. After a subsequent 8 minute incubation period, plates were read on a Synergy Neo2 plate reader (BioTek Instruments) at 20 ms/well and a read height of 1.0 mm. All incubations apart from antigen coating were performed at room temperature, and all plate washes were conducted using a 405 TS/LS LHC2 plate washer (BioTek Instruments); all wash steps included four washes with 100 µL PBS-T.

Blank-subtracted raw luminescence reads were normalized between dilutions by applying a scaling factor derived from the average ratios of the medians of on-plate controls. On-plate controls at 1:1,000 dilution were used as the reference point for normalization.

### Live ancestral SARS-CoV-2 neutralization assay

Neutralizing capacities of LTCH staff sera were assessed using live ancestral SARS-CoV-2 neutralization assays. LTCH staff serum samples were heat inactivated in a hot water bath at 56°C for 30 minutes and then allowed to cool to RT prior to use in live ancestral SARS-CoV-2 neutralization assays. All assays were conducted using Vero E6 cells (ATCC #CRL-1586) cultured in D-10 medium [Dulbecco's modified Eagle medium (DMEM) supplemented with 10% heat-inactivated FBS, 100 UI/mL penicillin, 100 UI/mL streptomycin, and 2 mM L-glutamine]. Briefly, 6 × 10^4^ Vero E6 cells were seeded per well of a 96-well flat bottom tissue culture plate and rested overnight at 37°C, 5% CO_2_. LTCH staff serum samples were then serially diluted in serum-free DMEM and incubated in the presence of 100 TCID_50_ of ancestral SARS-CoV-2 (SARS-CoV-2-SB2-PB clone 1) for 1 h at 37°C, 5% CO_2_. Following incubation, Vero E6 cells were inoculated with the virus/serum co-culture for 1 h at 37°C, 5% CO_2_. The inoculum was subsequently removed from all wells and replaced with D-2 medium (DMEM supplemented with 2% heat-inactivated FBS, 100 UI/mL penicillin, 100 UI/mL streptomycin, and 2 mM L-glutamine) before being incubated for 5 days at 37°C, 5% CO_2_. Following the incubation period, the cytopathic effect (CPE) of the viral inoculation was visually assessed by examining Vero E6 cells under a phase-contrast microscope. All live ancestral SARS-CoV-2 neutralization assays were conducted in quadruplicates. Results were analyzed using PRISM GraphPad software (GraphPad Software, La Jolla, CA). A 4-parameter logistic non-linear regression analysis was performed to compute 50% inhibitory concentration (IC_50_) values, which represent serum concentrations at which 50% of wells were negative for CPE. All handling of the live ancestral SARS-CoV-2 virus was performed within the Combined Containment Level 3 Unit at the Temerty Faculty of Medicine, University of Toronto.

All values of serological neutralizing capacity are provided as log(1/IC_50_). Sera with no neutralizing capacity were assigned a value of 0.

### Mathematical modeling approach and parameter estimation

A mathematical model of exponential growth (equation 1) or decay (equation 2) was fit to individual study data. All fits were performed in Monolix (version 2020R1) using non-linear mixed-effects models. For each data type (e.g., IFN-γ), we simultaneously fit all individuals to determine best-fit individual responses as well as a best-fit population response. Exponential growth/decay rates were determined for patients with increasing/decreasing measures of IFN-γ, IL-2, IgG, IgA, and log(1/IC_50_). For each fit, we computed random effect parameters (and relative standard error on random effects). Random effects are practical identifiability analyses to characterize the data-driven model identifiability outcomes. We further compute and provide the relative standard error on each set of fitted population parameters. The equations for growth and decay kinetics are,


(1) $$\frac{{dy}_{i}}{dt}=\mu_{i}y_{i}$$

(2) $$\frac{{dy}_{i}}{dt}=-\gamma_{i}y_{i},$$

where *i* corresponds to the *i ^th^* individual in the study, y corresponds to the clinical feature being fit (i.e., IFN-γ, IL-2, IgG, IgA, or log[1/IC_50_]), $\mu_{i}$ is the growth rate, and $\gamma_{i}$ is the decay rate.

### Statistical analysis

The non-parametric Kruskal-Wallis test (46) was employed to compute cross-sectional comparisons at similar timepoints between groups within categorizations of sex and vaccine types. The non-parametric Dunn’s test was employed to compute cross-sectional comparisons at similar timepoints between *n* = 5 immunological phenotype groups. *P*-values were corrected for multiple comparisons using the Benjamini-Hochberg method. The non-parametric Wilcoxon signed-rank test was employed to compute longitudinal comparisons from 2 to 6 weeks to 6 months post-second dose of BNT162b2 or mRNA-1273. * = 0.01 < *P* ≤ 0.05, ** = 0.001 < *P* ≤ 0.01, *** = 0.0001 < *P* ≤ 0.001, **** = *P* ≤ 0.0001. All correlations between spike-specific T-cell responses to mRNA vaccination and serological anti-spike/RBD IgG and IgA antibody levels or neutralizing capacity were computed using non-parametric Spearman’s tests (*r*_*s*_ = 0.00–0.19 [very weak]; *r*_*s*_ = 0.20–0.39 [weak]; *r*_*s*_ = 0.40–0.59 [moderate]; *r*_*s*_ = 0.60–0.79 [strong]; *r*_*s*_ = 0.80–1.0 [very strong]).

## RESULTS

### Sample acquisition

**Fig 1:**
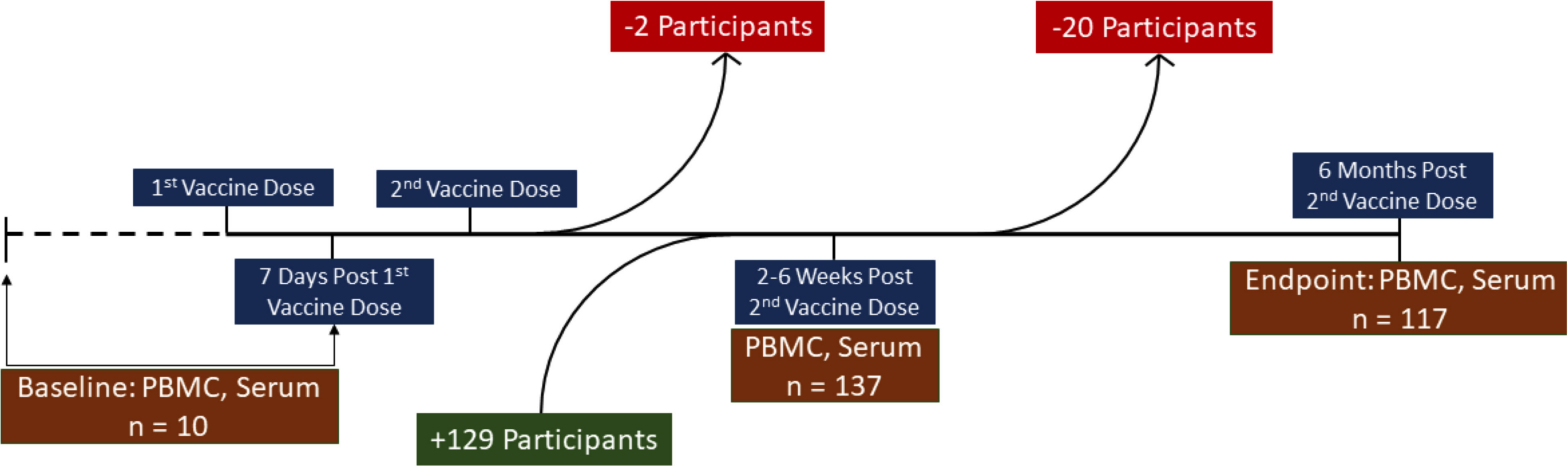
An overview of the LTCH staff cohort throughout the study.

To investigate how SARS-CoV-2-specific T-cell responses contribute to mRNA vaccine-induced humoral immunity, T-cell and humoral responses to BNT162b2 or mRNA-1273 were longitudinally assessed in a cohort of 139 high-risk LTCH staff. PBMC and serum samples were collected across three timepoints from 10 February to 14 December 2021: baseline (up to 7 days post-first dose, *n* = 10; low sample size due to rapid vaccine rollouts and delays in obtaining REB approval); 2–6 weeks post-second dose of BNT162b2 or mRNA-1273 (*n* = 137; 129 were additionally enrolled, 2 withdrew from baseline); and 6 months post-second dose (*n* = 117; 20 withdrew or were not analyzed because they received a third dose of mRNA vaccine). Refer to Fig. 1 for a graphical overview of the LTCH staff cohort throughout the study. According to Ontario public health records, the Alpha variant of SARS-CoV-2 predominated from February to late May 2021. Delta later became the major variant of concern from June to December 2021 (47–49).

### Immunological phenotypes of LTCH staff

To assess the effects of SARS-CoV-2-specific T-cell immunity on mRNA vaccine-induced humoral immunity, the following clinical features were used to stratify participants into separate immunological phenotypes: (i) history of previous PCR- or RAT-confirmed SARS-CoV-2 infections (see Materials and Methods); (ii) T-cell responses against SARS-CoV-2 N, E, M, S, and NSP masterpools by ELISpot at baseline or 2-6 weeks post-second dose; and (iii) anti-nucleocapsid (anti-N) IgG/IgA antibody levels in the serum.

**Fig 2:**
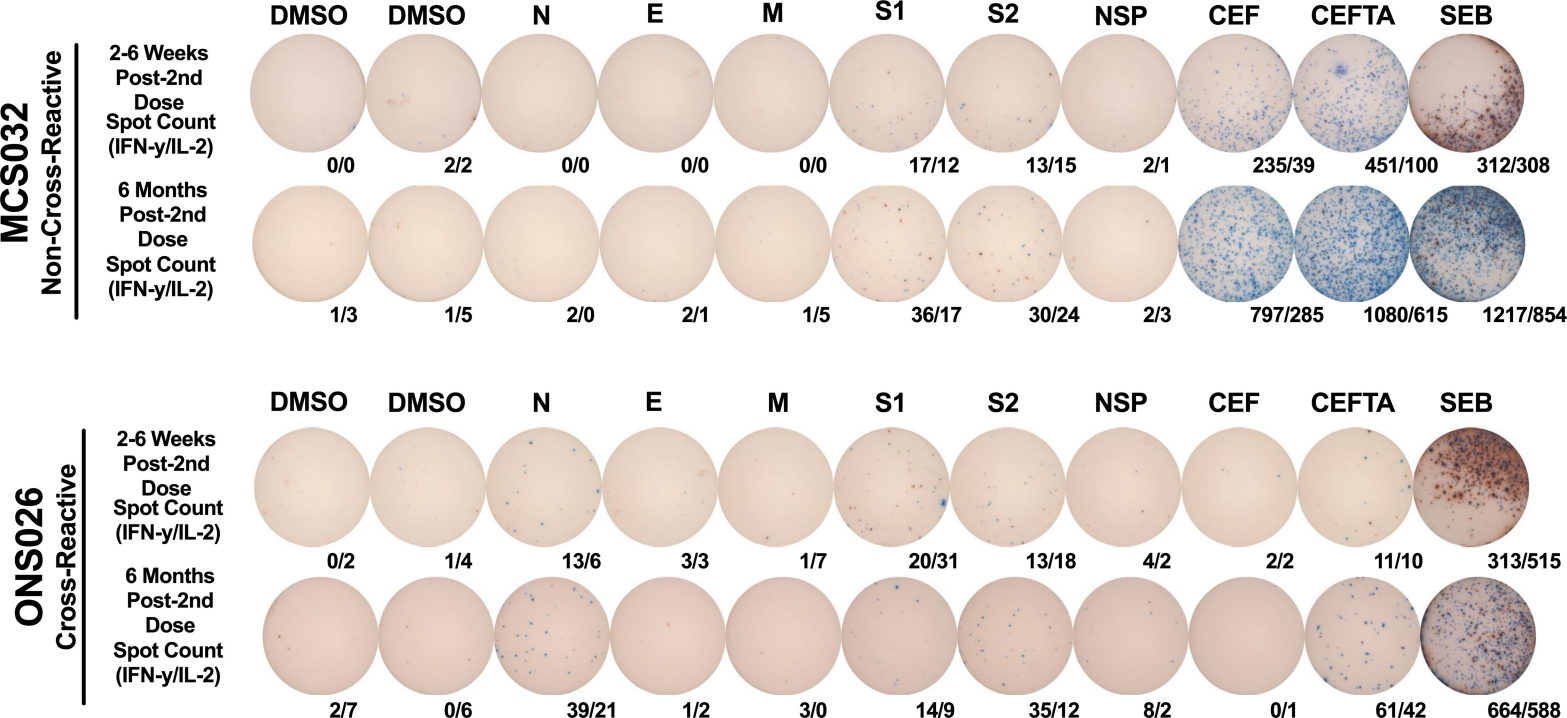
A representative ELISpot comparing spike and non-spike-specific IFN-γ (blue) and IL-2 (red) T-cell responses between non-cross-reactive and cross-reactive vaccinees. Spot counts are provided as IFN-γ/IL-2 spot-forming cells (SFC) per 250,000 PBMC. DMSO = negative controls; *N* = nucleocapsid; E = envelope; M = membrane; S1 + S2 = full spike; NSP = non-structural protein; CEF + CEFTA + SEB = positive controls.

Based on these clinical features, a total of five immunological phenotypes were defined as follows:

- Uninfected participants: Participants who consistently tested negative for SARS-CoV-2 by PCR/RAT and remained seronegative for anti-N IgG/IgA antibodies both prior to and during the study were denoted as “uninfected.” Uninfected participants could be further sub-stratified into two subgroups:
  - Non-T-cell cross-reactive (NCR): Those who failed to exhibit detectable IFN-γ or IL-2 T-cell responses by ELISpot to any SARS-CoV-2 masterpools at baseline or to any non-spike masterpools at 2–6 weeks post-second dose (Fig. 2).
  - T-cell cross-reactive (CR): Those who demonstrated detectable IFN-γ or IL-2 T-cell responses by ELISpot to any SARS-CoV-2 masterpool at baseline or to any non-spike masterpools at 2–6 weeks post-second dose (Fig. 2).
- Hybrid immune (HI): Participants who contracted a PCR/RAT-confirmed SARS-CoV-2 infection prior to either the first or second dose of BNT162b2 or mRNA-1273. Refer to Table S2 for a summary of clinical characteristics.
- Asymptomatic breakthrough: Participants who seroconverted to possess anti-N IgG/IgA antibodies between 2 and 6 weeks and 6 months post-second dose without ever displaying COVID-19-related symptoms or testing positive by PCR/RAT. Refer to Table S3 for a summary of clinical characteristics. No cases of symptomatic breakthrough infections were detected.
- Anti-N+, PCR(−): Participants who were already seropositive for anti-N IgG/IgA antibodies upon initial sample collection with no prior history of COVID-19-related symptoms or PCR/RAT-confirmed SARS-CoV-2 infections. It has been shown by previous literature that 20%–23% of pre-pandemic individuals—from as early as 2014—possessed serological titers of anti-N antibodies that were cross-reactive with SARS-CoV-2 (50, 51). Accordingly, since it was not possible to determine whether N+, PCR(−) participants identified at initial sample collection were convalescent or healthy with pre-existing humoral cross-reactivity, they were decidedly stratified into their own group.
- Unknown: Participants who could not be clearly categorized due to an absence of cellular or serological data. These individuals could not be included in any analyses.

Table 1 provides a summary of the clinical characteristics of the LTCH staff cohort at each timepoint of the study.

**TABLE 1:** Summary of clinical characteristics of the LTCH staff cohort at each timepoint

	Timepoint	Male/female	Average age (range in years)	Immunological phenotype					
				Uninfected		HI^*c*^	*N*+, PCR(−)^*d*^	Asymptomatic breakthrough^*e*^	Unknown
				NCR^*a*^	CR^*b*^				
	Baseline	3/7	43 (21–63)	1/10 (10%)	6/10 (60%)	3/10 (30%)	0/10 (0%)	–	0/10 (0%)
BNT162b2	2–6 Weeks post-second dose	10/77	45 (21–67)	21/87 (24%)	45/87 (52%)	11/87 (13%)	4/87 (5%)	–	6/87 (7%)
	6 Months post-second dose	10/64	45 (21–67)	17/74 (23%)	36/74 (49%)	10/74 (14%)	3/74 (4%)	5/74 (7%)	3/74 (4%)
mRNA-1273	2–6 Weeks post-second dose	7/43	49 (23–73)	15/50 (30%)	16/50 (32%)	13/50 (26%)	4/50 (8%)	–	2/50 (4%)
	6 Months post-second dose	7/36	49 (24–73)	13/43 (30%)	12/43 (28%)	10/43 (23%)	4/43 (9%)	3/43 (7%)	1/43 (2%)

^*a*^ Non-cross-reactive.

^*b*^ Cross-reactive.

^*c*^ Positive for anti-N IgG or IgA antibodies in serum, but negative for COVID-19 by PCR/RAT.

^*d*^ Hybrid immune.

^*e*^ Seroconverted to possess anti-N IgG/IgA antibodies between 2 and 6 weeks and 6 months post-second dose; remained asymptomatic.

### Comparing HCoV antibody levels between CR and NCR vaccine recipients

Given former evidence of the induction of cross-reactive T-cell immunity to SARS-CoV-2 by previous HCoV infections (34–37), we sought to determine whether CR vaccinees possessed elevated serological anti-spike IgG antibody levels against HCoV-HKU1, HCoV-NL63, HCoV-OC43, or HCoV-229E compared to NCR vaccine recipients at 2–6 weeks post-second dose. Antibody levels were observed to be highly similar between the two groups (Fig. S1).

### Comparing SARS-CoV-2 non-spike-specific T-cell responses between CR and HI vaccine recipients

As reported in previous studies, pre-existing cross-reactive T-cells in unexposed individuals frequently target regions of SARS-CoV-2 structural and non-structural antigens that are conserved between SARS-CoV-2 and the endemic HCoVs (34, 35, 37, 40, 52, 53). We thus investigated how the distribution of T-cell responses across non-spike antigens of SARS-CoV-2 compared between CR and HI vaccine recipients following the second dose of BNT162b2 or mRNA-1273. Anti-N+, PCR(−) (*n* = 7) participants were not included in these analyses due to a very small sample size.

**Fig 3:**
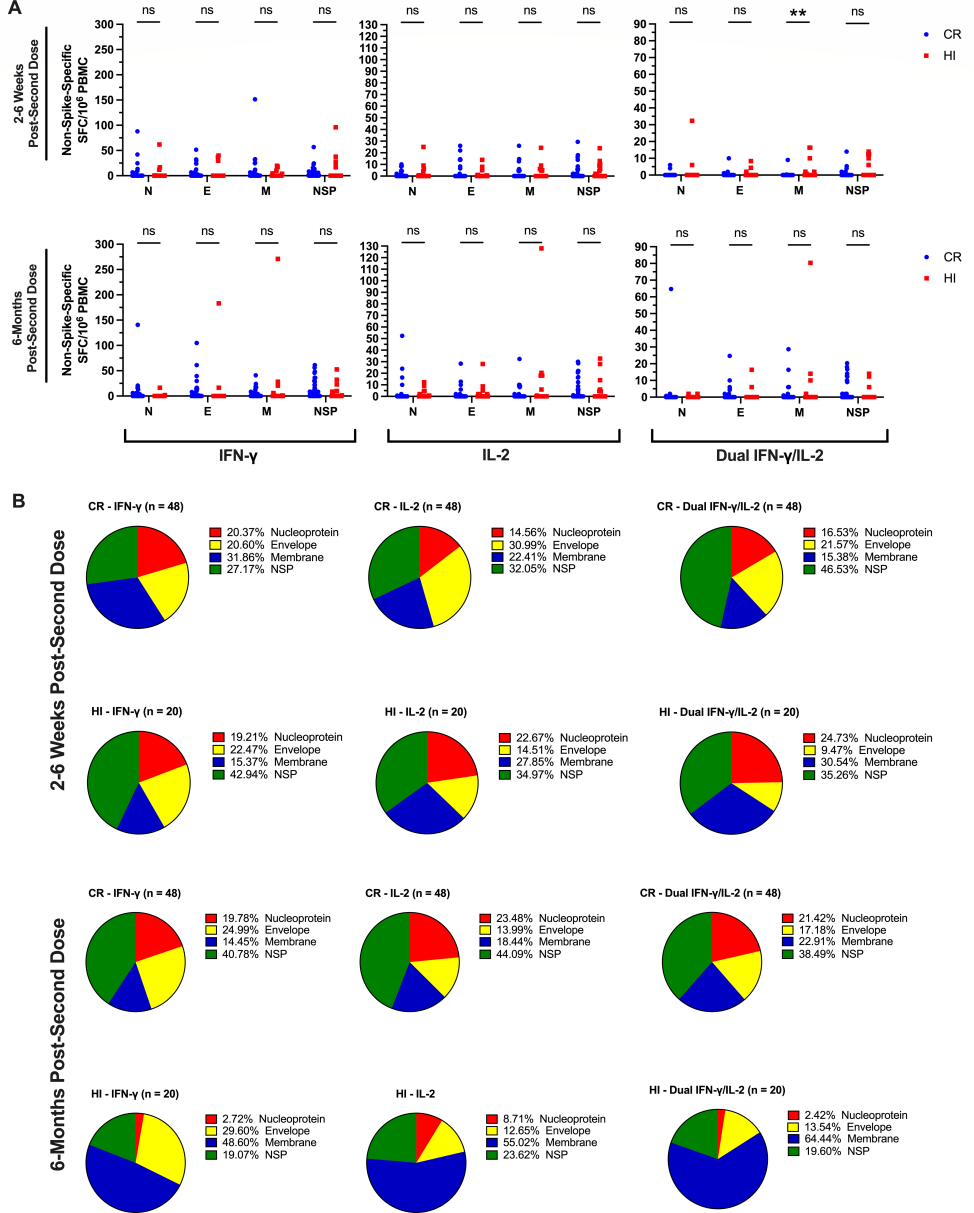
(**A**) Scatterplot comparisons of N-, E-, M-, and NSP-specific T-cell responses between CR (blue circles) and HI (red squares) vaccine recipients. (**B**) Pie chart comparisons of the distribution of IFN-γ, IL-2, and dual IFN-γ/IL-2 T-cell responses to SARS-CoV-2 nucleoprotein (red), envelope (yellow), membrane (blue), and NSP (green) peptide masterpools between HI and CR vaccine recipients of BNT162b2 or mRNA-1273 at 2–6 weeks and 6 months post-second dose.

As shown in Fig. 3A, vaccine recipients mounted significantly greater median dual IFN-γ/IL-2 T-cell responses against M compared to CR recipients at 2–6 weeks post-second dose (*P* = 0.007). Although differences were later lost by 6 months post-second dose (Fig. 3A), M-specific T-cell responses began to dominate the total non-spike-specific response in HI vaccine recipients (Fig. 3B). CR recipients contrarily began to mount the majority of non-spike specific T-cell responses against the NSP masterpool (Fig. 3B), which is comprised of 15-mer peptides with a considerable degree of homology with endemic HCoVs (34).

### Assessment of ancestral SARS-CoV-2 spike-specific T-cell responses in LTCH staff over the course of 6 months post-second dose of BNT162b2 or mRNA-1273

We next studied mRNA vaccine-induced spike-specific T-cell responses in LTCH staff following mRNA vaccination with BNT162b2 or mRNA-1273. Dual-color IFN-γ/IL-2 ELISpot assays were used to characterize these responses at 2–6 weeks and 6 months post-second dose. Mathematical models of exponential growth/decay were additionally fit to individual data points to analyze the precise kinetics of spike-specific T-cell responses across the two timepoints. Due to a scarcity of sample, ELISpot assays could only be conducted on whole PBMCs without any enrichment for the CD4^+^ T-cell subset.

**Fig 4:**
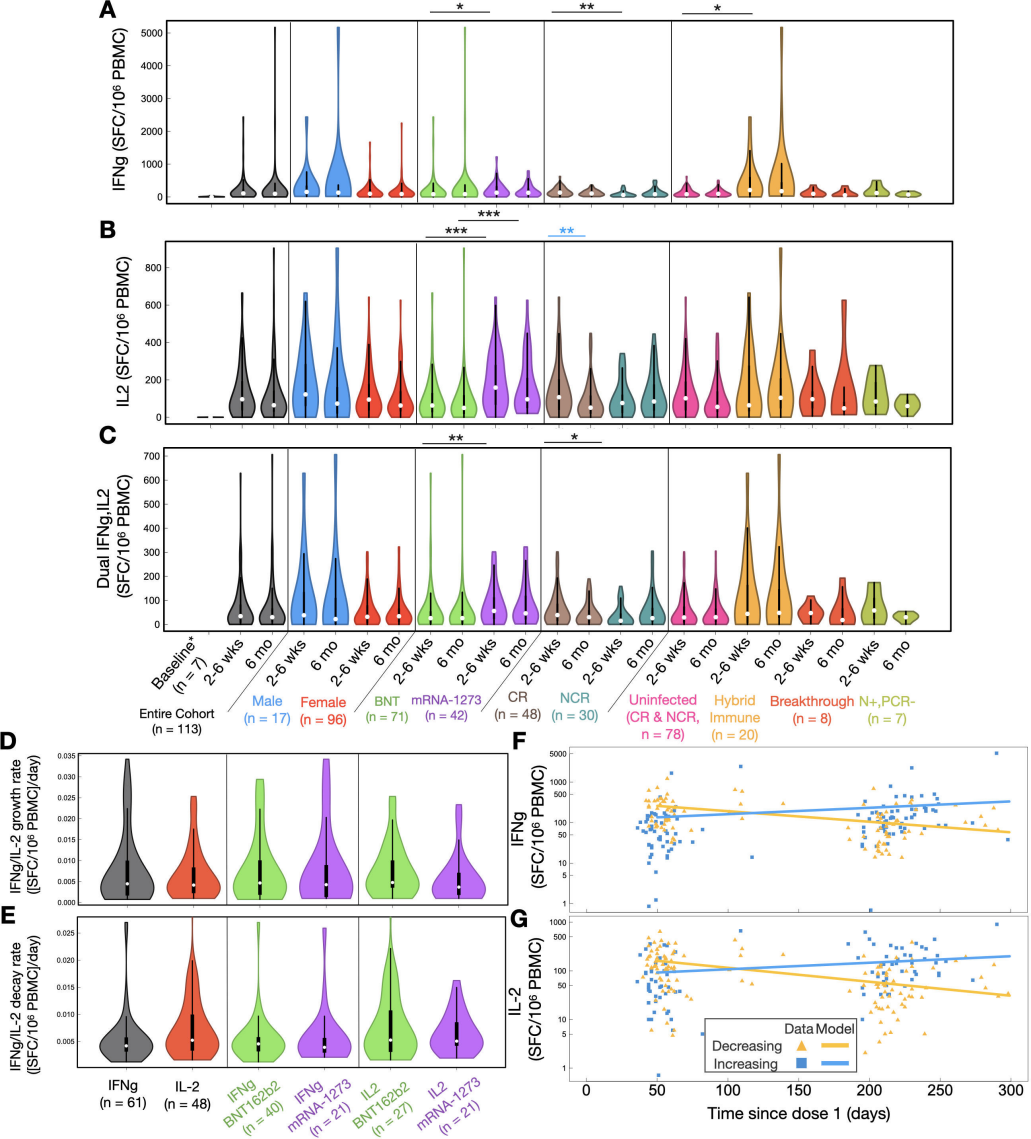
(**A–C**) comparisons of net spike-specific (**A**) IFN-γ, (**B**) IL-2, and (**C**) dual IFN-γ/IL-2 T-cell responses at 2–6 weeks and 6 months post-second dose. Squares and whiskers within each violin plot represent medians with interquartile ranges (IQRs). Refer to Table S4 for medians with IQRs for each group. Significant cross-sectional comparisons are indicated with black asterisks. Significant longitudinal comparisons are indicated with blue asterisks. Baseline is comprised of a group of seven uninfected LTCH staff. (**D, E**) Violin plot analysis of (**D**) growth and (**E**) decay rates in spike-specific IFN-γ and IL-2 T-cell responses across the entire cohort and between mRNA vaccine types. Squares and whiskers within each violin plot represent medians with IQRs. (**F, G**) Spike-specific, (**F**) IFN-γ, and (**G**) IL-2 T-cell responses as a function of time since dose 1 for *n* = 113 participants that could be analyzed longitudinally, separated by increasing (blue) and decreasing (yellow) trends. Refer to Table S5 for a summary of median decay and growth rates with half-lives and doubling times.

Relative to baseline, robust spike-specific IFN-γ, IL-2, and dual IFN-γ/IL-2 T-cell responses were observed in LTCH staff at 2–6 weeks post-second dose of BNT162b2 or mRNA-1273 (Fig. 4A through C; Table S4). Over the course of 6 months post-second dose, 60/113 (53%) LTCH staff analyzed unexpectedly exhibited a growth in spike-specific IFN-γ T-cell responses with an average doubling time of 155 (20–949) days (Fig. 4D and F). The remaining 53/113 (47%) had experienced a decay with an average half-life of 165 (26–567) days (Fig. 4E and F). With respect to spike-specific IL-2 T-cell responses, 48/113 (42%) LTCH staff likewise exhibited a growth with an average doubling time of 167 (27–715) days (Fig. 4D and G). The remaining 65/113 (58%) experienced a decay with an average half-life of 132 (22–436) days (Fig. 4E and G). Resultantly, median spike-specific IFN-γ and IL-2 T-cell responses across the entire cohort were not observed to significantly differ over the course of 6 months post-second dose (Fig. 4A through C).

Stratifying for vaccine type showed that recipients of mRNA-1273 generally exhibited significantly greater spike-specific T-cell responses than those of BNT162b2 at 2–6 weeks post-second dose (Fig. 4A through C). However, the durability of the response—defined by the rate of decline overtime—was not impacted by the mRNA vaccine platform (Fig. 4D and E). Stratifying for sex additionally did not uncover any differences in vaccine-induced spike-specific T-cell responses (Fig. 4A through C) or durability (data not shown) between males and females.

To assess the influence of pre-existing T-cell cross-reactivity to SARS-CoV-2 on mRNA vaccine-induced spike-specific T-cell responses, uninfected participants were stratified into NCR and CR vaccine recipients. In comparison to NCR vaccinees, CR recipients of BNT162b2 or mRNA-1273 exhibited a significant boost in spike-specific IFN-γ (*P* = 0.0014) and dual IFN-γ/IL-2 (*P* = 0.034) T-cell responses at 2–6 weeks post-second dose (Fig. 4A and C). However, these boosts were transient and observed to dissipate by 6 months post-second dose (Fig. 4A and C). CR vaccine recipients additionally experienced significant declines (*P* = 0.0038) in spike-specific IL-2 T-cell responses by 6 months post-second dose, which were contrastingly entirely preserved in NCR vaccine recipients (Fig. 4B).

Comparing uninfected participants to all other immunological phenotypes revealed significantly greater spike-specific IFN-γ T-cell responses in HI vaccinees (*P* = 0.048) at 2–6 weeks post-second dose (Fig. 4A).

### Assessment of serological anti-spike and anti-RBD IgG and IgA antibody levels over a period of 6 months post-second dose of BNT162b2 or mRNA-1273

ELISAs measuring levels of anti-spike and anti-RBD IgG and IgA antibody levels in sera were deployed to characterize humoral responses induced by mRNA vaccination in LTCH staff at 2–6 weeks and 6 months post-second dose. As before, mathematical models of exponential grow/decay were fit to individual data points for the analysis of response kinetics across the two timepoints.

**Fig 5:**
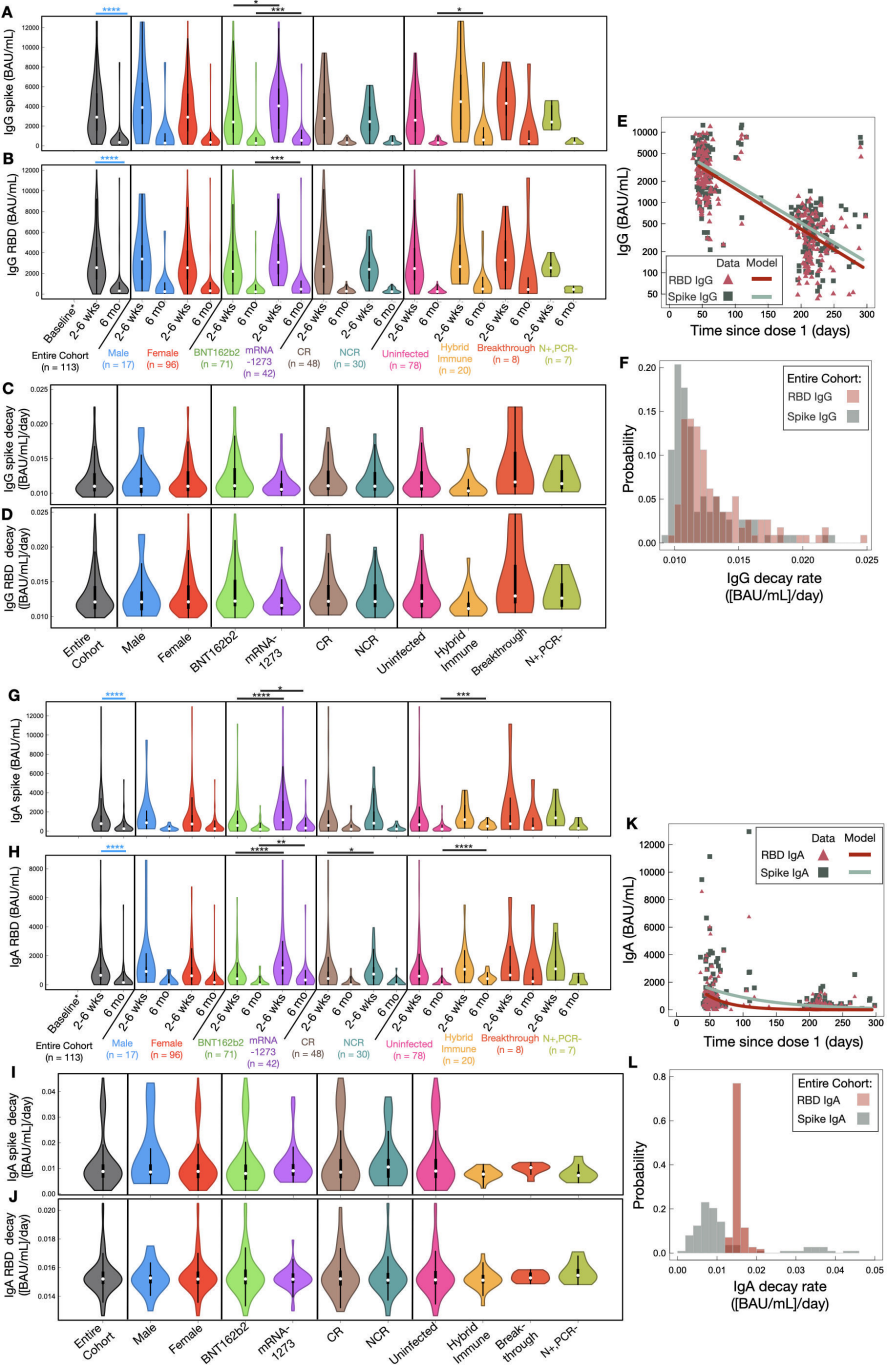
(**A, B, G, H**) Violin plot analysis of serological (**A**) anti-spike IgG, (**B**) anti-RBD IgG, (**G**) anti-spike IgA, and (**H**) anti-RBD IgA antibody levels at 2–6 weeks and 6 months post-second dose. Squares and whiskers within each violin plot represent medians with IQRs (see Table S6 for medians with IQRs). Significant cross-sectional comparisons are indicated with black asterisks. Significant longitudinal comparisons are displayed with blue asterisks for the entire cohort alone to avoid overcrowding the figure. Baseline is comprised of a group of seven uninfected LTCH staff. (**C, D, I, J**) Violin plot analysis of (**C**) anti-spike IgG, (**D**) anti-RBD IgG, (**I**) anti-spike IgA, and (**J**) anti-RBD IgA decay rates across the entire LTCH staff cohort after adjusting for sex, vaccine type, and immunological phenotype. (**E, K**) Mathematical modeling of decay rates in (**E**) anti-spike IgG (green), anti-RBD IgG (red), (**K**) anti-spike IgA (green), and anti-RBD IgA (red) antibody levels since dose 1 of BNT162b2 or mRNA-1273. (**F, L**) A histogram of individual decay rates in (**F**) anti-spike IgG (green), anti-RBD IgG (red), (**L**) anti-spike IgA (green), and anti-RBD IgA (red) antibody levels. Refer to Table S7 for a summary of median decay rates with half-lives for anti-spike and anti-RBD IgG and IgA antibody levels.

Relative to baseline, robust anti-spike and anti-RBD IgG antibody levels were observed across the entire LTCH staff cohort at 2–6 weeks post-second dose of BNT162b2 or mRNA-1273 (Fig. 5A and B; Table S6). However, over the course of 6 months post-second dose, 111/113 (98.5%) LTCH staff exhibited highly significant decays (*P* < 0.0001) in anti-spike and anti-RBD IgG antibody levels with approximate half-lives of 63 (31–73) days and 57 (28–71) days, respectively, (Fig. 5A through F).

Compared to recipients of BNT162b2, anti-spike IgG antibody levels were significantly elevated (*P* = 0.0279) in mRNA-1273 vaccinees at 2–6 weeks post-second dose (Fig. 5A). Despite similar decay kinetics (Fig. 5C and D), differences were later accentuated by 6 months post-second dose and both anti-spike (*P* = 0.0002) and anti-RBD (*P* = 0.0008) IgG antibody levels became greater in mRNA-1273 vaccinees (Fig. 5A and B).

Stratifying for sex did not uncover differences in the magnitude or durability of anti-spike/RBD IgG antibody levels between males and females (Fig. 5A through D).

Pre-existing cross-reactive T-cell immunity to SARS-CoV-2 did not significantly boost mRNA vaccine-induced anti-spike or anti-RBD IgG antibody levels in uninfected vaccine recipients (Fig. 5A and B). As shown in Fig. 5C and D and Table S7, the durability of anti-spike and anti-RBD IgG antibodies were also highly similar between NCR and CR vaccine recipients as well as across all other immunological phenotypes. However, anti-spike IgG antibody levels were significantly greater in HI vaccine recipients compared to uninfected recipients at 6 months post-second dose (*P* = 0.029; Fig. 5A).

With respect to anti-spike and anti-RBD IgA antibody levels, all LTCH staff possessed robust levels in the serum at 2–6 weeks post-second dose (Fig. 5G and H; Table S6). As with IgG, 98.5% of participants exhibited a significant decline in anti-spike and anti-RBD IgA antibody levels (*P* < 0.0001) over the course of 6 months post-second dose with approximate half-lives of 79 (15–526) days and 46 (34–55) days, respectively, (Fig. 5G–L). Of importance to note, a 2–6 week window post-second dose may not have captured the initial rapid decay of IgA antibodies and may have thus overestimated half-life measures compared to IgG (26, 44, 54, 55).

Stratifying by vaccine type showed that recipients of mRNA-1273 had significantly higher anti-spike and anti-RBD IgA antibody levels than those of BNT162b2 recipients (*P* < 0.0001) at 2–6 weeks post-second dose (Fig. 5G and H). Given that decay rates were highly similar between the two groups (Fig. 5I and J; Table S7), anti-spike (*P* = 0.0160) and anti-RBD (*P* = 0.0013) IgA antibody levels remained greater in mRNA-1273 vaccinees at 6 months post-second dose.

As before, sex was not observed to influence the magnitude or durability of spike- and RBD-specific IgA antibody levels (Fig. 5G–J).

When stratifying our cohort by immunological phenotype, NCR vaccine recipients were observed to possess significantly greater anti-RBD IgA antibody levels (*P* = 0.044) than CR recipients at 2–6 weeks post-second dose (Fig. 5H). However, differences between the two groups were transient and dissipated by 6 months post-second dose (*P* = 0.37; Fig. 5H). No differences in anti-spike IgA antibody levels were observed between NCR and CR vaccine recipients at either timepoint (Fig. 5G).

With respect to all other immunological phenotype groups, no differences in anti-spike or anti-RBD IgA antibody levels were observed at 2–6 weeks post-second dose (Fig. 5G and H). However, by 6 months post-second dose, anti-spike and anti-RBD IgA antibody levels in HI vaccine recipients became higher than in uninfected participants (*P* = 0.0009 and *P* < 0.0001, respectively; Fig. 5G and H).

### Assessment of serological neutralization of live ancestral SARS-CoV-2 over a period of 6 months post-second dose

We lastly conducted live ancestral SARS-CoV-2 neutralization assays to characterize the neutralizing capacity of LTCH staff sera at 2–6 weeks and 6 months post-second dose. Mathematical models of exponential growth/decay were again fitted to individual values to evaluate response kinetics over time.

**Fig 6:**
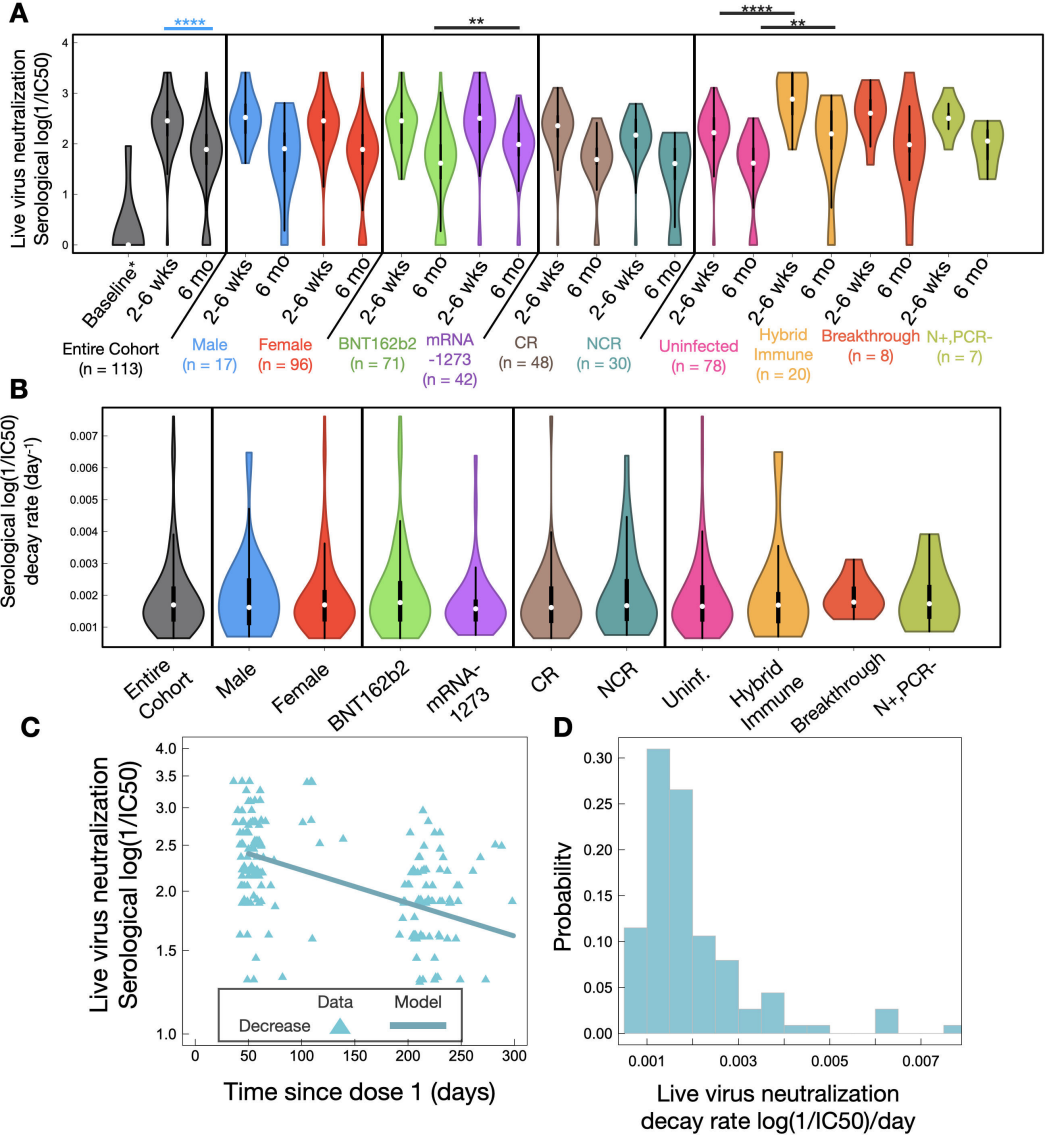
(**A**) Violin plot analysis of serological neutralizing capacity against ancestral SARS-CoV-2 at 2–6 weeks and 6 months post-second dose. Squares and whiskers within each violin plot represent medians with IQRs. Refer to Table S8 for medians with IQRs for each group. Significant cross-sectional comparisons are indicated with black asterisks. Significant longitudinal comparisons are displayed with blue asterisks for the entire cohort alone to avoid overcrowding the figure. Baseline is comprised of a group of seven uninfected LTCH staff. (**B**) Violin plot analysis of decay rates in serological neutralizing capacity across the entire LTCH staff cohort and following adjustments for sex, vaccine type, and immunological phenotype. (**C**) Mathematical modeling of decay in serological neutralizing capacity since dose 1 of BNT162b2 or mRNA-1273. (**D**) A histogram of individual decay rates in serological neutralizing capacity. Refer to Table S9 for a summary of median decay rates with half-lives.

Across the entire cohort, LTCH staff sera had largely demonstrated robust neutralizing capacity against ancestral SARS-CoV-2 at 2–6 weeks post-second dose of BNT162b2 or mRNA-1273 (Fig. 6A). However, with the wane of SARS-CoV-2 spike- and RBD-specific IgG and IgA antibody levels, serological neutralizing capacity likewise exhibited significant declines over the course of 6 months post-second dose (*P* < 0.0001; Fig. 6A). The half-life of the entire cohort was computed to be 408 (91–1,063) days.

Stratifying for sex revealed no statistically significant difference in durability (Fig. 6B; Table S9) or median serological neutralizing capacity (Fig. 6A) between males and females.

Upon stratifying for vaccine type, the serological neutralization of live ancestral SARS-CoV-2 trended to be greater in recipients of mRNA-1273 than BNT162b2 at 2–6 weeks post-second dose, although statistical significance was not quite attained (*P* = 0.0838; Fig. 6A). However, despite similar decay kinetics between the two vaccine groups (Fig. 6B; Table S9), serological neutralizing capacity later became significantly greater in recipients of mRNA-1273 (*P* = 0.0021) at 6 months post-second dose (Fig. 6A).

Assessing the impacts of pre-existing T-cell cross-reactivity to SARS-CoV-2 on serological neutralizing capacity showed that CR vaccine recipients trended to exhibit greater neutralization than NCR recipients (*P* = 0.057) at 2–6 weeks post-second dose (Fig. 6A). However, durability was not observed to differ between the two groups (Fig. 6B; Table S9) and differences were diminished by 6 months post-second dose (Fig. 6A).

HI vaccinees were lastly observed to exhibit superior serological neutralizing capacity compared to uninfected participants at both 2–6 weeks (*P* < 0.0001) and 6 months (*P* = 0.0056) post-second dose (Fig. 6A).

### Correlations between spike-specific T-cell and humoral responses to mRNA vaccination with BNT162b2 or mRNA-1273

Having now characterized the cellular and humoral immune responses induced by mRNA vaccination, we next assessed how spike-specific IFN-γ and IL-2 T-cell responses in LTCH staff may have contributed to the magnitude and durability of vaccine-induced neutralizing antibody levels. To avoid potential confounding effects of natural infection, uninfected and hybrid immune participants were analyzed separately.

**Fig 7:**
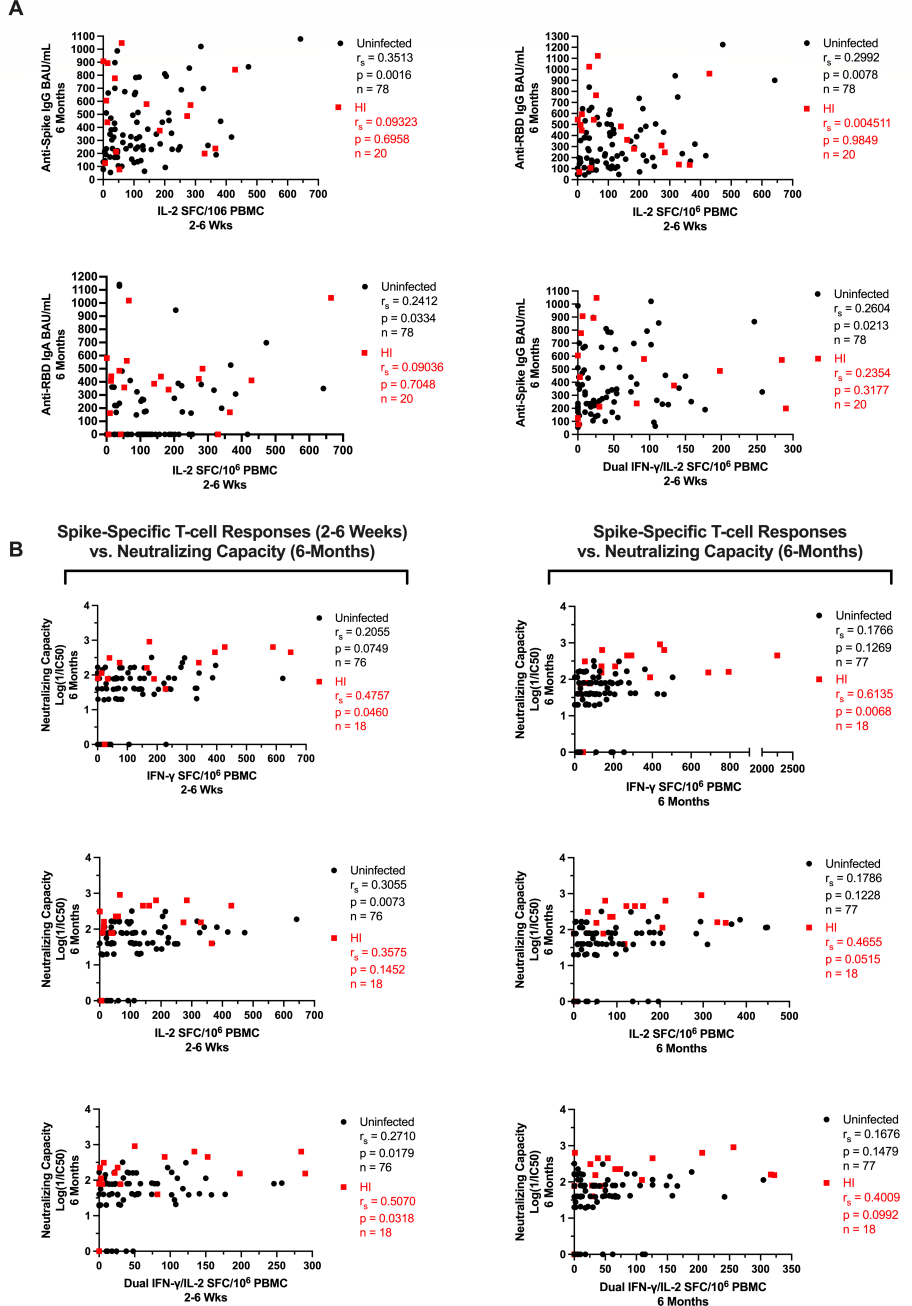
(**A**) Spike-specific T-cell responses at 2–6 weeks post-second dose plotted against serological anti-spike IgG, anti-RBD IgG, and anti-RBD IgA antibody levels at 6 months post-second in uninfected and HI LTCH staff. (**B**) Serological neutralizing capacity at 6 months plotted against spike-specific T-cell responses at 2–6 weeks or 6 months post-second dose. Black circles = uninfected; red squares = HI.

Notably, moderate to very strong positive correlations were observed between anti-spike and anti-RBD IgG/IgA antibody levels and serological neutralizing capacity. These relationships were generally strongest at 2–6 weeks post-second dose for HI vaccine recipients and at 6 months post-second dose for uninfected recipients (Fig. S2). Accordingly, we first investigated how mRNA vaccine-induced spike-specific T-cell responses correlated with serological antibody levels at either timepoint. No correlations could be detected at either 2–6 weeks or 6 months post-second dose. However, within uninfected participants, initial spike-specific IL-2 T-cell responses at 2–6 weeks post-second dose were found to weakly correlate with serological anti-spike IgG (*r*_*s*_ = 0.3513; *P* = 0.0016), anti-RBD IgG (*r*_*s*_ = 0.2992; *P* = 0.0078), and anti-RBD IgA (*r*_*s*_ = 0.2412; *P* = 0.0334) antibody levels at the later 6 month timepoint (Fig. 7A). Initial dual IFN-γ/IL-2 T-cell responses to spike at 2–6 weeks were likewise observed to weakly correlate with later anti-spike IgG antibody levels (*r*_*s*_ = 0.2604; *P* = 0.0213) at 6 months post-second dose (Fig. 7A). No such correlations could be observed for HI vaccine recipients (Fig. 7A).

We next investigated whether spike-specific T-cell responses directly correlated with serological neutralizing capacity. Within the HI vaccinee group, moderate positive correlations were observed between initial spike-specific IFN-γ (*r*_*s*_ = 0.4757, *P* = 0.0460) and dual IFN-γ/IL-2 (*r*_*s*_ = 0.5070, *P* = 0.0318) T-cell responses at 2–6 weeks post-second dose and serological neutralizing capacity at 6 months post-second dose (Fig. 7B). Contrastingly, within uninfected vaccinees, only weak positive correlations could be detected between initial spike-specific IL-2 (*r*_*s*_ = 0.3055; *P* = 0.0073) and dual IFN-γ/IL-2 (*r*_*s*_ = 0.2710; *P* = 0.0179) T-cell responses at 2–6 weeks post-second dose and serological neutralizing capacity at 6 months post-second dose (Fig. 7B).

Moreover, at 6 months post-second dose, spike-specific IFN-γ T-cell responses were observed to strongly correlate (*r*_*s*_ = 0.6135, *P* = 0.0068) with serological neutralizing capacity in HI vaccine recipients (Fig. 7B). Moderate positive correlations approaching significance were additionally observed for spike-specific IL-2 (*r*_*s*_ = 0.4655, *P* = 0.0515) and dual IFN-γ/IL-2 (*r*_*s*_ = 0.4009, *P* = 0.0992) T-cell responses (Fig. 7B). However, no correlations could be observed for uninfected vaccine recipients at this timepoint (Fig. 7B).

### Potential off-target T-cell responses induced in vaccine recipients of BNT162b2 or mRNA-1273

Throughout the study, IFN-γ, IL-2, and dual IFN-γ/IL-2 non-spike-specific T-cell responses were continuously monitored by ELISpot in LTCH staff. In the following analyses, these non-spike-specific T-cell responses are reported as cumulative measures of nucleocapsid, envelope, membrane, and NSP-specific responses.

**Fig 8:**
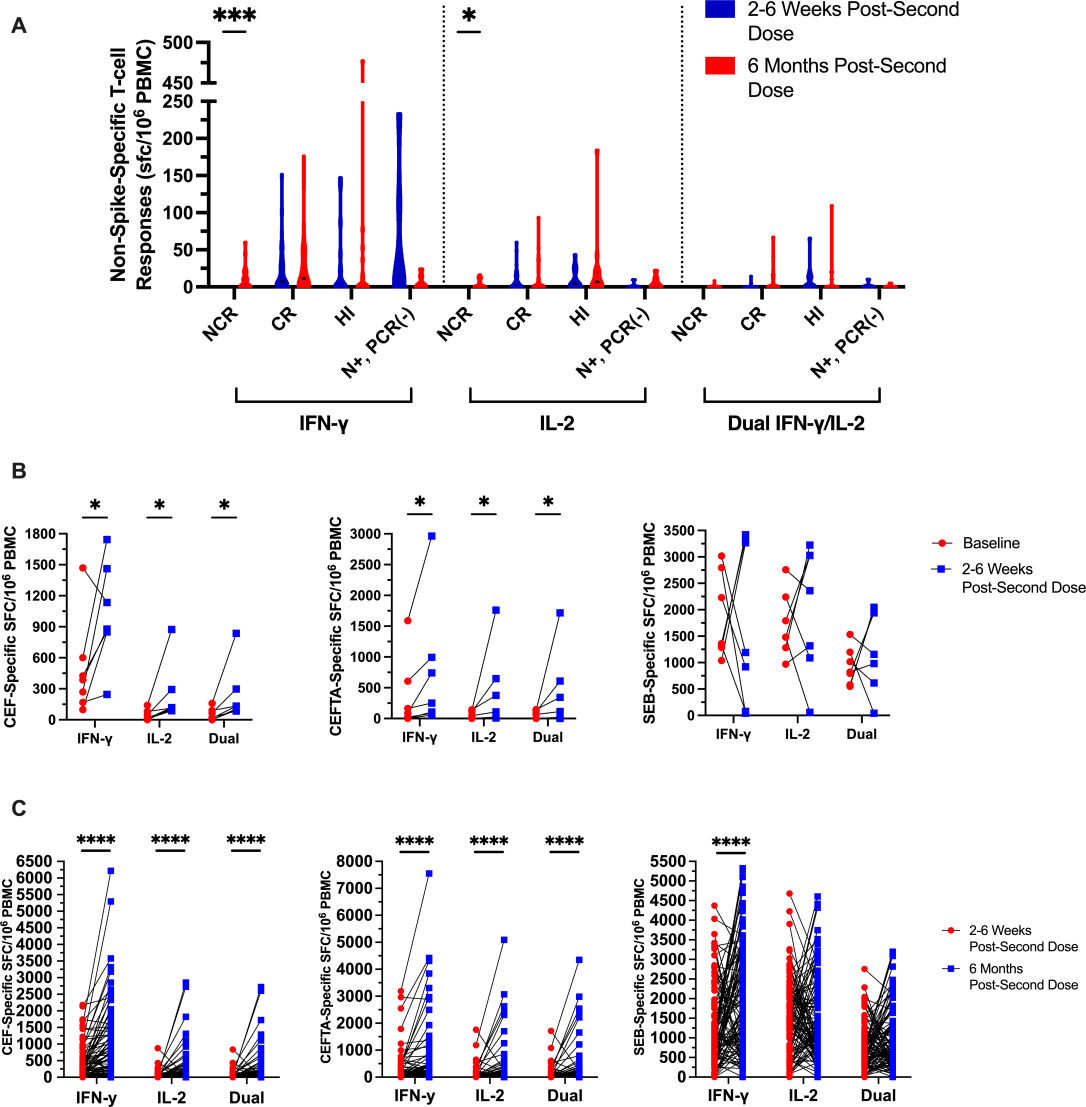
A longitudinal comparison of net non-spike-specific IFN-γ, IL-2, and dual IFN-γ/IL-2 T-cell responses from 2 to 6 weeks to 6 months post-second dose in NCR (*n* = 30), CR (*n* = 48), HI (*n* = 20), and N+, PCR(−) (*n* = 7) vaccine recipients. Non-spike-specific T-cell responses represent cumulative responses to nucleoprotein, envelope, membrane, and non-structural protein. Violin plots represent median responses with interquartile ranges. (**B**) Longitudinal pairwise comparisons (*n* = 7) of net CEF-, CEFTA-, and SEB-specific IFN-γ, IL-2, and dual IFN-γ/IL-2 T-cell responses from baseline (red circles) to 2–6 weeks post-second dose (blue squares). (**C**) Longitudinal pairwise comparisons (*n* = 105) of net CEF-, CEFTA-, and SEB-specific IFN-γ, IL-2, and dual IFN-γ/IL-2 T-cell responses from 2 to 6 weeks (red circles) to 6 months post-second dose (blue squares).

From 2–6 weeks to 6 months post-second dose, trending increases in non-spike-specific T-cell responses were noticed in the majority of vaccine recipients assessed (Fig. 8A). Most notably, 21/30 (70%) NCR vaccine recipients began exhibiting non-spike-specific IFN-γ (*P* = 0.0004) and IL-2 (*P* = 0.0107) T-cell responses at 6 months post-second dose without ever reporting any clinical symptoms compatible with COVID-19, testing positive for COVID-19 by PCR/RAT, or seroconverting to possess anti-N IgG or IgA antibodies (Fig. 8A; Fig. S3). This may suggest that a portion of NCR participants identified at 2–6 weeks post-second dose had possessed pre-existing cross-reactive T-cell memory to SARS-CoV-2 that was below detectable levels but later amplified by off-target vaccine effects.

To investigate this possibility, we first conducted longitudinal comparisons in CEF-, CEFTA-, and SEB-specific IFN-γ, IL-2, and dual IFN-γ/IL-2 T-cell responses from baseline to 6 months post-second dose. As shown in Fig. 8B, baseline participants (*n* = 7) had generally exhibited significant increases in CEF- and CEFTA-specific IFN-γ, IL-2, and dual IFN-γ/IL-2 T-cell responses by 2–6 weeks post-second dose (*P* = 0.0461). However, no significant changes in SEB-specific T-cell responses could be observed (Fig. 8B).

Increases in both CEF- and CEFTA-specific T-cell responses became most striking when comparisons were made between 2–6 weeks and 6 months post-second dose (*P* < 0.0001; Fig. 8C). Highly significant increases were likewise observed for SEB-specific IFN-γ (*P* < 0.0001), but not IL-2 or dual IFN-γ/IL-2 T-cell responses (Fig. 8C). This was predominantly observed for participants who exhibited increases in spike-specific T-cell responses from 2 to 6 weeks to 6 months post-second dose. Positive control responses were otherwise generally stabilized among recipients who experienced declines in spike-specific T-cell responses overtime. These observations were not accompanied by any significant longitudinal changes in negative control responses (data not shown). To address potential batch effects, we repeated assays on leftover 2–6 week and 6 month post-second dose timepoint samples from two participants that were processed on separate dates and replicated our original findings (data not shown).

We next investigated whether undetectable non-spike-specific T-cell responses in NCR recipients at 2–6 weeks post-second dose could be amplified *in vitro*. For this experiment, four NCR recipients were randomly selected. Given that all participants in our study were previously exposed to HCoV (Fig. S1), our highly conserved NSP masterpool (34) was used to screen for undetectable T-cell cross-reactivity at 2–6 weeks post-second dose. Briefly, PBMC samples were expanded for 7 days under stimulation with NSP, CEF, CEFTA, or SEB. T-cell responses were then assessed against these antigens by ELISpot (see Materials and Methods). Although the 7 day *in vitro* expansion had amplified CEF-, CEFTA-, and SEB-specific IFN-γ and IL-2 T-cell responses in all four NCR recipients, NSP-specific T-cell responses only became detectable in 1/4 (25%) recipients at 2–6 weeks post-second dose (data not shown).

## DISCUSSION

T cells are key players in the adaptive immune response to invading pathogens and are essential for the development of neutralizing antibody titers (12, 15, 16). While several previous studies have evaluated the associations between SARS-CoV-2-specific CD4^+^ T-cell responses, serological antibody titers, and neutralization within the context of natural infection (4, 5, 20–22), very few studies to date have evaluated these associations within the context of vaccination (23, 24). The impact of pre-existing cross-reactive T-cell immunity to SARS-CoV-2 has additionally only been investigated in a few studies (33), and its precise effects on the kinetics of cellular and humoral responses to vaccination are yet unclear. The aim of this study was to thoroughly investigate the associations between mRNA vaccine-induced spike-specific IFN-γ and IL-2 T-cell responses and neutralizing antibody development. The impacts of pre-existing cross-reactive T-cell immunity to SARS-CoV-2 on the magnitude and kinetics of cellular and humoral responses to vaccination were also investigated.

We began our study by first characterizing T-cell and humoral responses induced by mRNA vaccination with BNT162b2 or mRNA-1273 in our LTCH staff cohort. As expected, relative to baseline, robust spike-specific IFN-γ and IL-2 T-cell responses, anti-spike/RBD IgG and IgA antibody levels, and serological neutralization of ancestral SARS-CoV-2 were observed at 2–6 weeks post-second dose. In line with previous studies, although vaccine-induced humoral responses significantly waned over the course of 6 months post-second dose, spike-specific T-cell responses across the entire cohort were well preserved (23, 24, 56–61). Recipients of mRNA-1273 were generally observed to exhibit significantly greater spike-specific T-cell responses, anti-spike IgG/IgA antibody levels, and serological neutralizing capacity than those of BNT162b2, which likewise agrees with multiple previous studies (62–65). Mathematical modeling of T-cell response kinetics over the course of 6 months post-second dose revealed a gradual growth of spike-specific IFN-γ and IL-2 T-cell responses in 53% and 42% of vaccinees, respectively. A gradual decrease was contrastingly observed in all remaining vaccine recipients. To our knowledge, no previous studies have reported similar findings. However, a plausible explanation may be “cellular sensitization without seroconversion,” whereby SARS-CoV-2-specific T-cell responses are expanded in response to viral exposure in the absence of seroconversion, COVID-19-related symptoms, or positive PCR testing (66–68). The likelihood of this phenomenon for our study is increased by the fact that our LTCH staff cohort worked and lived in high-risk conditions during the COVID-19 pandemic.

In uninfected vaccine recipients, weak positive correlations were observed between initial spike-specific IL-2 T-cell responses at 2–6 weeks and serological neutralizing capacity at 6 months post-second dose. A previous study has similarly demonstrated significant positive correlations between mRNA vaccine-induced spike-specific IL-2 T-cell responses 2–4 weeks post-second dose and neutralizing antibody levels 3–4 months post-second dose in individuals with immune-mediated inflammatory diseases and in healthy controls (24). These correlations became stronger in HI vaccine recipients where spike-specific IFN-γ or dual IFN-γ/IL-2 T-cell responses at 2–6 weeks correlated with greater serological neutralizing capacity at 6 months post-second dose (Fig. 7B). Spike-specific IFN-γ T-cell responses moreover become strongly associated with neutralizing capacity within the latter timepoint (Fig. 7B). Greater spike-specific IFN-γ T-cell responses in HI vaccinees thus likely improved vaccine durability and contributed to greater neutralizing capacity at 6 months post-second dose compared to uninfected participants. Previous studies have similarly reported enhanced durability in neutralizing antibody levels in HI recipients after a two-dose mRNA vaccine regimen (69, 70).

Due to sample scarcities, a limitation of our study was our inability to distinguish between CD4^+^ and CD8^+^ T-cell responses and our measurement of only two cytokines (IFN-γ and IL-2) by ELISpot. However, previous studies have shown that the T-cell response to SARS-CoV-2 is predominantly CD4^+^ and characterized by IFN-γ, tumor necrosis factor-alpha (TNFα), and IL-2 (6, 71, 72). mRNA vaccination against SARS-CoV-2 was likewise found to induce a CD4^+^ T-cell dominant response, with IL-2 being primarily secreted by CD4^+^ T-cells and IFN-γ being secreted by a mix of CD4^+^ and CD8^+^ T-cells (73). Thus, IL-2 responses in our study may be interpreted to primarily represent the CD4^+^ T-cell population, whereas IFN-γ likely reflects contributions from both T-cell subsets. The superior sensitivity of the ELISpot assay also enabled us to detect lower frequency responses to spike and other antigens that would have otherwise been missed by alternative assays including flow cytometry.

We next investigated how pre-existing T-cell cross-reactivity to SARS-CoV-2 may have impacted the magnitude and kinetics of T-cell and humoral responses induced by mRNA vaccination. Within our study, CR vaccinees were a sub-stratification of uninfected participants who exhibited significant spike-specific T-cell responses by ELISpot to any SARS-CoV-2 masterpool at baseline or to any non-spike masterpool at 2–6 weeks post-second dose. We understood that cellular sensitization without seroconversion may potentially explain the non-spike-specific T-cell responses exhibited by CR vaccine recipients. A comparison between HI and CR vaccinees showed that CR recipients predominantly mounted non-spike-specific T-cell responses against the NSP masterpool at 6 months post-second dose. These observations strongly aligned with Swadling et al. who reported a preferential expansion of T-cell responses against NSP7 (contained within our NSP masterpool) and NSP12—highly conserved antigenic targets across human common-cold coronaviruses—in exposed seronegative healthcare workers who repeatedly tested negative for COVID-19 by PCR (66). Akin to their study, we additionally observed a preferential expansion of T-cell responses toward the membrane masterpool in HI vaccine recipients, which is contrastingly less conserved across human common-cold coronaviruses (66). Accordingly, with regard to non-spike-specific T-cell responses after vaccination, CR vaccinees were phenotypically distinct from HI recipients.

CR recipients exhibited significant boosts in spike-specific IFN-γ and dual IFN-γ/IL-2 T-cell responses at 2–6 weeks post-second dose compared to NCR vaccinees. Yet, consistent with a previous study (38), the boosts were transient and seemingly dissipated by 6 months post-second dose. In agreement with reference 39, we also could not detect significant differences in anti-spike/RBD IgG antibody levels between CR and NCR vaccine recipients. Although serological neutralizing capacity trended to be greater in CR vaccinees at 2–6 weeks post-second dose, differences were lost by 6 months post-second dose. This contrasted with reference 39, which continuously detected elevated serological neutralizing capacity in cross-reactive vaccine recipients by 6 months post-second dose. Discrepancies may be explained by the administration of a lower 25 µg dose of mRNA-1273 in reference 39 compared to the standard 100 µg dose administered in our study. A higher 100 µg dose of mRNA-1273 may have elicited a more potent response that masked the effects of pre-existing cross-reactivity on neutralizing capacity. Our pooling of BNT162b2 and mRNA-1273 participants in our analysis may have also diminished our ability to detect differences between NCR and CR vaccinees. Nonetheless, pre-existing T-cell cross-reactivity to SARS-CoV-2 was not observed to impact the kinetics of mRNA vaccine-induced spike-specific IgG responses or serological neutralizing capacity.

Anti-RBD IgA levels were transiently lower in CR than in NCR vaccine recipients at 2–6 weeks post-second dose. This may be attributable to the greater spike-specific IFN-γ T-cell responses observed in CR vaccinees at this timepoint—recent literature suggests significant associations between heightened type I and type II interferon signaling and delayed anti-RBD antibody kinetics in COVID-19 patients (74). CR vaccine recipients were additionally the only immunological phenotype to have experienced significant declines in spike-specific IL-2 T-cell responses from 2 to 6 weeks to 6 months post-second dose. Whether this is due to the emigration of cells from the circulation to specific tissues (75, 76) requires further study.

From 2–6 weeks to 6 months post-second dose, most vaccine recipients exhibited trending increases in non-spike-specific T-cell responses to SARS-CoV-2. These increases were especially prominent and had reached statistical significance within NCR vaccine recipients. Within NCR recipients, we hypothesized that the emergence of non-spike-specific T-cell responses to SARS-CoV-2 at 6 months post-second dose was attributable to the vaccine-induced amplification of previously undetectable responses. Of four NCR vaccinees tested at 2–6 weeks post-second dose, T-cell responses to our conserved NSP peptide masterpool could only be amplified to detectable levels in one recipient. However, given that all recipients in our study exhibited evidence of exposure to previous common-cold coronaviruses (Fig. S1), it is still likely that the remaining 3/4 NCR recipients tested possessed cross-reactive T-cell memory to SARS-CoV-2 that was not captured using our NSP peptide masterpool.

Highly significant increases in CEF-, CEFTA-, and SEB-specific T-cell responses were additionally observed across the entire LTCH staff cohort by 6 months post-second dose, especially among individuals who exhibited increases in spike-specific T-cell responses throughout the study. This may be due to bystander activation or trained immunity, which reportedly induce off-target immune responses to unrelated antigens following vaccination or natural infection (77–85). Consistent with trained immunity, Föhse et al. (81) reported significant increases in IL-6 and IL-1β responses to heat-killed SARS-CoV-2, *Candida albicans*, and *Staphylococcus aureus* at 6 months post-first dose (5.5 months post-second dose) of BNT162b2 compared to baseline (81). Relative to a first dose, Arunachalam et al. (85) had additionally observed elevated frequencies of CD14+CD16+ inflammatory monocytes and heightened plasma concentrations of IFN-γ following a second dose of BNT162b2 (85). By extension, consistent with the possibility of bystander activation, Epstein Barr virus (EBV) and cytomegalovirus (CMV) reactivation, which may occur in response to non-specific immune activation (86–89), has been noted in some patients following mRNA vaccination (90–92). The induction of bystander activation and trained immunity following mRNA vaccination thus warrants further investigation in future studies.

In summary, our data highlight the potential contributions of mRNA vaccine-induced SARS-CoV-2 spike-specific T-cell responses to serological neutralizing capacity within both uninfected and HI vaccine recipients up to 6 months post-second dose. Pre-existing cross-reactive T-cell immunity to SARS-CoV-2 had only a modest effect on the magnitude and kinetics of T-cell and humoral responses to mRNA vaccination. These findings should help inform the development of next-generation T cell-based coronavirus vaccines and vaccine regimens that can minimize the impacts of both current and future human coronaviruses of concern.

## Data Availability

All data supporting the findings of this study are included within the article and its supplemental material.

## SUPPLEMENTAL MATERIAL

Supplemental tables and figures jvi.01685-24-s0001.docx Tables S1 to S9; Fig. S1 to S3.

ASM does not own the copyrights to Supplemental Material that may be linked to, or accessed through, an article. The authors have granted ASM a non-exclusive, world-wide license to publish the Supplemental Material files. Please contact the corresponding author directly for reuse.
