# Supplementary material for: mRNA vaccine-induced SARS-CoV-2 spike-specific IFN-γ and IL-2 T-cell responses are predictive of serological neutralization and are transiently enhanced by pre-existing cross-reactive immunity
Source: J Virol. 2025 Jan 31;99(3):e01685-24. doi: 10.1128/jvi.01685-24 (PMC11915849; doi:10.1128/jvi.01685-24)
Supplement: Supplemental tables and figures — Tables S1 to S9; Fig. S1 to S3. [file jvi.01685-24-s0001.docx]

**SUPPLEMENTARY MATERIALS**

**Table S1:** 15-mer peptides constituting the NSP masterpool.

| **Sequence** | **Organism** | **Protein** | **Start** |
| --- | --- | --- | --- |
| MFHLVDFQVTIAEIL | SARS-CoV-2 | ORF6 | 1 |
| TFKVSIWNLDYIINL | SARS-CoV-2 | ORF6 | 21 |
| SDFVRATATIPIQAS | SARS-CoV-2 | ORF3a | 26 |
| YIINLIIKNLSKSLT | SARS-CoV-2 | ORF6 | 31 |
| FYSKWYIRVGARKSA | SARS-CoV-2 | ORF8 | 41 |
| ALLAVFQSASKIITL | SARS-CoV-2 | ORF3a | 51 |
| VKHVYQLRARSVSPK | SARS-CoV-2 | ORF7a | 71 |
| PLNSIIKTIQPRVEK | SARS-CoV-2 | nsp2 | 276 |
| EEIAIILASFSASTS | SARS-CoV-2 | nsp2 | 471 |
| SPLYAFASEAARVVR | SARS-CoV-2 | nsp2 | 531 |
| QTFFKLVNKFLALCA | SARS-CoV-2 | nsp2 | 676 |
| KVTFFPDLNGDVVAI | SARS-CoV-2 | nsp3 | 1956 |
| KHFYWFFSNYLKRRV | SARS-CoV-2 | nsp4 | 3151 |
| NHNFLVQAGNVQLRV | SARS-CoV-2 | nsp5 | 3326 |
| NRYFRLTLGVYDYLV | SARS-CoV-2 | nsp6 | 3801 |
| VLKKLKKSLNVAKSE | SARS-CoV-2 | nsp8 | 3976 |
| KLLKSIAATRGATVV | SARS-CoV-2 | nsp12 | 4966 |
| EFYAYLRKHFSMMIL | SARS-CoV-2 | nsp12 | 5136 |
| LRKHFSMMILSDDAV | SARS-CoV-2 | nsp12 | 5141 |
| LMIERFVSLAIDAYP | SARS-CoV-2 | nsp12 | 5246 |
| TSHKLVLSVNPYVCN | SARS-CoV-2 | nsp13 | 5361 |
| ISPYNSQNAVASKIL | SARS-CoV-2 | nsp13 | 5836 |
| NVNRFNVAITRAKVG | SARS-CoV-2 | nsp13 | 5881 |
| REEAIRHVRAWIGFD | SARS-CoV-2 | nsp14 | 6001 |
| TQLCQYLNTLTLAVP | SARS-CoV-2 | nsp16 | 6846 |
| QIDGYVMHANYIFWR | SARS-CoV-2 | nsp16 | 7016 |

**Table S2:** Clinical characteristics of hybrid immune LTCH staff analyzed.

| **Participant ID** | **# of PCR-Confirmed Infections** | **Date of Most Recent Positive PCR Test**  **(yyyy-mm-dd)** | **Date of 1^st^ Vaccine Dose** | **Date of 2^nd^ Vaccine Dose** | **Days between Infection and 1^st^ Dose** | **Days between Infection and 2^nd^ Dose** | **Severity of Infection^[[1]](#footnote-1)^** | **Date of Blood Draw 2-6 Weeks Post-Second Dose**  **(yyyy-mm-dd)** |
| --- | --- | --- | --- | --- | --- | --- | --- | --- |
| **RSS002** | 1 | 2020-04-07 | 2021-01-03 | 2021-01-31 | -271 | -299 | 2 | 2021-02-20 |
| **CVS001** | 1 | 2020-04-25 | 2021-01-06 | 2021-01-27 | -256 | -277 | 2 | 2021-02-11 |
| **CCS004** | 1 | 2020-05-22 | 2021-01-19 | 2021-02-16 | -242 | -270 | 2 | 2021-03-10 |
| **CWS001** | 1 | 2020-09-25 | 2021-03-17 | 2021-02-25 | -173 | -153 | 2 | 2021-03-18 |
| **VSS009** | 1 | 2020-10-01 | 2021-02-25 | 2021-05-19 | -147 | -230 | 1 | 2021-06-15 |
| **DLS019** | 1 | 2020-11-02 | 2021-03-25 | 2021-06-03 | -143 | -213 | 1 | 2021-06-23 |
| **VSS007** | 1 | 2020-10-13 | 2021-01-18 | 2021-02-15 | -97 | -125 | 1 | 2021-03-11 |
| **VSS004** | 1 | 2020-10-01 | 2021-01-05 | 2021-02-02 | -96 | -124 | 1 | 2021-02-20 |
| **VSS003** | 1 | 2020-10-18 | 2021-01-10 | 2021-02-15 | -84 | -120 | 2 | 2021-03-02 |
| **VSS008** | 1 | 2020-10-16 | 2021-01-05 | 2021-02-02 | -81 | -109 | 2 | 2021-03-02 |
| **ONS010** | 1 | 2021-01-11 | 2021-03-15 | 2021-06-04 | -63 | -144 | 2 | 2021-06-28 |
| **NLS010** | 1 | 2021-01-10 | 2021-02-21 | 2021-05-22 | -42 | -132 | 2 | 2021-06-10 |
| **CVS002** | 1 | 2020-11-25 | 2021-01-04 | 2021-02-01 | -40 | -68 | 1 | 2021-02-18 |
| **NLS003** | 1 | 2021-01-13 | 2021-02-20 | 2021-05-21 | -38 | -128 | 2 | 2021-06-10 |
| **CVS004** | 1 | 2021-01-04 | 2021-01-04 | 2021-02-01 | 0 | -28 | 2 | 2021-02-18 |
| **DLS018** | 1 | 2021-01-06 | 2021-01-04 | 2021-01-25 | 2 | -19 | 0 | 2021-03-02 |
| **CVS003** | 1 | 2021-01-17 | 2021-01-13 | 2021-02-17 | 4 | -31 | 1 | 2021-03-11 |
| **CCS006** | 1 | 2021-02-10 | 2021-01-19 | 2021-02-26 | 22 | -16 | 1 | 2021-03-18 |
| **MCS035** | 1 | 2021-01-20 | 2020-12-28 | 2021-02-05 | 23 | -16 | 2 | 2021-03-04 |
| **DLS013^[[2]](#footnote-2)^** | 1 | 2021-02-09 | 2021-01-05 | 2021-01-26 | 35 | 14 | 0 | 2021-02-20 |

**Table S3**: A summary of clinical characteristics of breakthrough vaccinees following infection.

| **Participant ID** | **Age** | **Sex** | **Vaccine Received** | **Clinical Characteristics Following Breakthrough Infection** | | | | **Immunological phenotype Prior to Breakthrough** |
| --- | --- | --- | --- | --- | --- | --- | --- | --- |
|  |  |  |  | **PCR/RAT(+)?** | **Anti-N IgG/IgA Seropositive?** | | **Reported Symptoms** |  |
| **CVS005** | 60 | Female | BNT162b2 | No | Yes | | None | Cross-Reactive |
| **CVS009** | 61 | Female | mRNA-1273 | No | Yes | | None | Cross-Reactive |
| **DPS006** | 49 | Female | BNT162b2 | No | Yes | | None | Non-Cross-Reactive |
| **DPS014** | 47 | Female | BNT162b2 | No | Yes | | None | Cross-Reactive |
| **DPS017** | 39 | Female | mRNA-1273 | No | Yes | | None | Cross-Reactive |
| **KGS011** | 49 | Female | mRNA-1273 | No | Yes | | None | Hybrid Immune |
| **MCS016** | 35 | Female | BNT162b2 | No | Yes | | None | Cross-Reactive |
| **MCS027** | 51 | Female | BNT162b2 | No | Yes | | None | Non-Cross-Reactive |
| **Breakthrough Rates** | | | | | | | | |
| **BNT162b2:** | | | 5/71 (7%) | **Uninfected** | | Non-Cross-Reactive: | | 2/32 (6%) |
| **mRNA-1273:** | | | 3/42 (7%) |  |  | Cross-Reactive: | | 5/53 (9%) |
| **Fisher’s Exact Test:** | | | **p > 0.9999** | **Hybrid Immune:** | | | | 1/21 (5%) |
|  | | | | **Fisher’s Exact Test Comparisons** | | | | |
|  |  |  |  | Non-Cross-Reactive vs. Cross-Reactive: | | | | **p = 0.7057** |
|  |  |  |  | Uninfected vs. Hybrid Immune: | | | | **p > 0.9999** |

**Table S4:** Spike-specific IFN-γ, IL-2, and dual IFN-γ/IL-2 T-cell responses following mRNA vaccination at 2-6 weeks and 6-months post-second dose.

|  | **Cytokine** | **Median Net Spike-Specific sfc/10^6^ PBMC (IQR)** | | | | | | | | | |
| --- | --- | --- | --- | --- | --- | --- | --- | --- | --- | --- | --- |
|  |  | **Entire Cohort** | **Sex** | | **Vaccine Type** | | **Immunological phenotype** | | | | |
|  |  |  | Male  (n = 17) | Female  (n = 96) | BNT162b2 (n = 71) | mRNA-1273  (n = 42) | **Uninfected** | | HI  (n = 20) | Breakthrough (n = 8) | N+  PCR(-)  (n = 7) |
|  |  |  |  |  |  |  | CR  (n = 48) | NCR  (n = 30) |  |  |  |
| **Baseline**  **(n = 7)** | **IFN-γ** | 0  (2.88) | - | - | - | - | - | - | - | - | - |
|  | **IL-2** | 0  (0.172) | - | - | - | - | - | - | - | - | - |
|  | **Dual**  **IFN-γ/IL-2** | 0  (0.00) | - | - | - | - | - | - | - | - | - |
| **2-6 Weeks Post 2^nd^ Dose (n = 113)** | **IFN-γ** | 106 (192.81) | 151.74 (279.89) | 95  (195.60) | 82  (154.03) | 128 (260.6) | 131.17 (178.32) | 59.09 (65.83) | 208.86 (538.24) | 93.52  (100) | 118 (147.14) |
|  | **IL-2** | 96.34 (154.44) | 122.16 (223.66) | 94.52 (140.72) | 62.94 (102.35) | 158.66 (212.43) | 107.17 (162.99) | 76.16 (95.88) | 64 (243.85) | 96.69  (89.60) | 84.1 (131.53) |
|  | **Dual**  **IFN-γ/IL-2** | 34.1 (74.56) | 38 (64.51) | 31.52 (76) | 26  (49.72) | 56 (103.06) | 38.89 (71.44) | 14.98 (43) | 44 (157.93) | 47.35  (29) | 58 (86.53) |
| **6-Months Post**  **2^nd^ Dose (n = 113)** | **IFN-γ** | 95.08  (147.31) | 128.69 (115.31) | 87.69 (147.48) | 85.03  (127.49) | 125.72 (200.69) | 102.86 (126.26) | 80.86 (115.50) | 174.68 (380.47) | 51.37  (87.20) | 72.12 (70.74) |
|  | **IL-2** | 64.34 (112) | 73.03 (140.35) | 62.69 (106.31) | 50  (99.35) | 96 (159.60) | 51.17 (95.92) | 84.34 (138.67) | 104 (159.77) | 47.69  (52.45) | 60 (46.32) |
|  | **Dual**  **IFN-γ/IL-2** | 30  (56) | 22 (120.97) | 34  (52) | 26  (54.49) | 46 (73.91) | 30 (45.92) | 26  (57) | 48 (121.33) | 18  (59.74) | 30 (18.80) |

**Table S5:** A summary of median growth and decay rates in spike-specific T-cell responses in LTCH staff from 2-6 weeks to 6-months post-second dose of BNT162b2 or mRNA-1273.

|  | | **IFN-**γ | | | **IL-2** | | |
| --- | --- | --- | --- | --- | --- | --- | --- |
|  |  | **Entire Cohort (n = 113)** | **BNT162b2**  **(n = 71)** | **mRNA-1273**  **(n = 42)** | **Entire Cohort (n = 113)** | **BNT162b2**  **(n = 71)** | **mRNA-1273**  **(n = 42)** |
| **Growth** | **Median Growth (d^-1^)** | 0.0045 | 0.0047 | 0.0043 | 0.0042 | 0.0048 | 0.0037 |
|  | **Growth IQR (d^-1^)** | 0.0083 | 0.0007 | 0.0008 | 0.0061 | 0.0023 | 0.0018 |
|  | **Doubling Time (d)** | 155.42 | 148.70 | 162.77 | 166.61 | 145.24 | 188.38 |
|  | **Doubling Time Range Max** | 948.69 | 948.69 | 719.09 | 715.12 | 715.12 | 711.14 |
|  | **Doubling Time Range Min** | 20.26 | 23.58 | 20.26 | 27.38 | 27.38 | 29.68 |
|  | **Relative Standard Error on Pop. Fit (%)** | 16.37 | - | - | 14.23 | - | - |
| **Decay** | **Median Decay (d^-1^)** | 0.0042 | 0.0045 | 0.0039 | 0.0052 | 0.0052 | 0.0051 |
|  | **Decay IQR (d^-1^)** | 0.0026 | 0.0026 | 0.0027 | 0.0066 | 0.0076 | 0.0043 |
|  | **Half-Life (d)** | 165.63 | 152.51 | 177.25 | 132.68 | 132.03 | 137.12 |
|  | **Half-Life Range Max** | 566.92 | 566.92 | 332.7 | 436.13 | 436.13 | 360.29 |
|  | **Half-Life Range Min** | 25.72 | 25.72 | 26.76 | 21.53 | 21.53 | 42.69 |
|  | **Relative Standard Error on Pop. Fit (%)** | 21.33 | - | - | 11.09 | - | - |

**Table S6:** Serological anti-spike IgG, anti-RBD IgG, anti-spike IgA, and anti-RBD IgA antibody levels following mRNA vaccination at 2-6 weeks and 6-months post-second dose.

|  | **Serum Antibody Titer** | **Median Binding Antibody Units/mL (IQR)** | | | | | | | | | |
| --- | --- | --- | --- | --- | --- | --- | --- | --- | --- | --- | --- |
|  |  | **Entire Cohort** | **Sex** | | **Vaccine Type** | | **Immunological phenotype** | | | | |
|  |  |  | Male  (n = 17) | Female  (n = 96) | BNT162b2 (n = 71) | mRNA-1273  (n = 42) | Uninfected | | HI  (n = 20) | Breakthrough (n = 8) | N+  PCR(-)  (n = 7) |
|  |  |  |  |  |  |  | CR  (n = 48) | NCR  (n = 30) |  |  |  |
| **Baseline**  **(n = 7)** | **Anti-Spike IgG** | 0  (0) | - | - | - | - | - | - | - | - | - |
|  | **Anti-RBD IgG** | 0  (0) | - | - | - | - | - | - | - | - | - |
|  | **Anti-Spike IgA** | 0  (0) | - | - | - | - | - | - | - | - | - |
|  | **Anti-RBD IgA** | 0  (0) | - | - | - | - | - | - | - | - | - |
| **2-6 Weeks Post 2^nd^ Dose (n = 113)** | **Anti-Spike IgG** | 2906.67  (3781.65) | 2505.39  (2907.2) | 3102.83  (4051.13) | 2300.48  (3812.06) | 4286.55  (3545.32) | 2782.39  (4228.77) | 2450.12  (2450.70) | 4480.59  (5561.67) | 4307.73  (2821.82) | 2411.78  (2020.76) |
|  | **Anti-RBD IgG** | 2647.62  (3112.48) | 2407.07  (2976.3) | 2717.89  (3120.18) | 2226.36  (3116.17) | 3124.05  (2761.25) | 2833.38  (3559.87) | 2391.16  (1639.18) | 2658.85  (3588.56) | 3295.86  (2931.96) | 2541.24  (935.79) |
|  | **Anti-Spike IgA** | 779.27  (1185.64) | 874.77  (521.36) | 741.83  (1226.67) | 589.73  (631.20) | 1335.88  (2703.35) | 592.03  (729.88) | 827.02  (1603.41) | 1193.39  (1964.25) | 764.31  (1187.97) | 1379.3  (1429.67) |
|  | **Anti-RBD IgA** | 648.84  (862.39) | 759.15  (591.33) | 599.07  (875.43) | 418.85  (476.72) | 1208.68  (1178.73) | 426.04  (665.48) | 730.71  (822.63) | 1062.45  (686.79) | 660.76  (911.28) | 1094.16  (1294.33 |
| **6-Months Post 2^nd^ Dose (n = 113)** | **Anti-Spike IgG** | 341.56  (454.29) | 284.61  (358.12) | 344.77  (457.10) | 257.67  (271.33) | 523.64  (493.89) | 320  (260.76) | 261.23  (332.40) | 592.44  (601.40) | 451.93  (550.91) | 287.04  (322.05) |
|  | **Anti-RBD IgG** | 293.05  (399.50) | 247.23  (357.42) | 333.56  (402.27) | 216.62  (316.97) | 468.69  (402.23) | 270.27  (310.56) | 224.96  (336.64) | 489.36  (542.97) | 439.49  (595.11) | 449.19  (471.56) |
|  | **Anti-Spike IgA** | 249.74  (376.54) | 263.33  (169.73) | 247.14  (379.24) | 204.61  (320.49) | 305.88  (379.10) | 192.87  (220.24) | 216.06  (209.90) | 513.95  (532.68) | 288.33  (476.15) | 296.19  (503.67) |
|  | **Anti-RBD IgA** | 148.34  (360.57) | 199.81  (410.26) | 74.17  (358.11) | 0  (244.93) | 313.12  (419.94) | 0  (224.89) | 0  (271.95) | 417.43  (217.33) | 224.90  (353.68) | 0  (595.53) |

**Table S7:** A summary of median decay rates in serological anti-spike and anti-RBD IgG and IgA antibody levels in vaccinated LTCH staff from 2-6 weeks to 6-months post-second dose of BNT162b2 or mRNA-1273.

|  | | **Entire Cohort (n = 113)** | **Sex** | | **Vaccine Type** | | **Immunological phenotype** | | | | |
| --- | --- | --- | --- | --- | --- | --- | --- | --- | --- | --- | --- |
|  |  |  | Male  (n = 17) | Female (n = 96) | BNT162b2 (n = 71) | mRNA-1273  (n = 42) | **Uninfected** | | HI  (n = 20) | Breakthrough (n = 8) | N+, PCR (-)  (n = 7) |
|  |  |  |  |  |  |  | CR  (n = 48) | NCR  (n = 30) |  |  |  |
| **Anti-Spike IgG** | **Median Decay (d^-1^)** | 0.0110 | 0.0110 | 0.0110 | 0.0111 | 0.0106 | 0.0111 | 0.0110 | 0.0103 | 0.0116 | 0.0114 |
|  | **Decay IQR (d^-1^)** | 0.0026 | 0.0022 | 0.0028 | 0.0031 | 0.0012 | 0.0028 | 0.0026 | 0.0008 | 0.0051 | 0.0027 |
|  | **Half-Life (d)** | 62.98 | 63.04 | 62.96 | 62.54 | 65.68 | 62.58 | 62.95 | 67.06 | 59.65 | 60.88 |
|  | **Half-Life Range Max** | 73.33 | 72.12 | 73.33 | 73.33 | 71.71 | 71.71 | 73.33 | 72.12 | 70.04 | 67.88 |
|  | **Half-Life Range Min** | 30.88 | 35.62 | 30.88 | 30.88 | 37.35 | 35.62 | 37.35 | 42.14 | 30.88 | 44.69 |
|  | **Relative Standard Error on Pop. Fit (%)** | 3.47 | - | - | - | - | - | - | - | - | - |
| **Anti-RBD IgG** | **Median Decay (d^-1^)** | 0.0121 | 0.0121 | 0.0121 | 0.0122 | 0.0116 | 0.0122 | 0.0122 | 0.0111 | 0.0130 | 0.0126 |
|  | **Decay IQR (d^-1^)** | 0.0033 | 0.0027 | 0.0034 | 0.0038 | 0.0017 | 0.0030 | 0.0032 | 0.0011 | 0.0055 | 0.0033 |
|  | **Half-Life (d)** | 57.31 | 57.31 | 57.24 | 56.73 | 59.77 | 56.91 | 57.02 | 62.24 | 53.51 | 54.84 |
|  | **Half-Life Range Max** | 70.51 | 68.83 | 70.51 | 70.51 | 67.79 | 67.79 | 70.51 | 68.83 | 65.63 | 62.57 |
|  | **Half-Life Range Min** | 28.01 | 31.80 | 28.01 | 28.01 | 34.73 | 31.80 | 34.73 | 37.69 | 28.01 | 39.68 |
|  | **Relative Standard Error on Pop. Fit (%)** | 4.08 | - | - | - | - | - | - | - | - | - |
| **Anti-Spike IgA** | **Median Decay (d^-1^)** | 0.0087 | 0.0085 | 0.0087 | 0.0079 | 0.0090 | 0.0085 | 0.0105 | 0.0077 | 0.0102 | 0.0072 |
|  | **Decay IQR (d^-1^)** | 0.0051 | 0.0041 | 0.0053 | 0.0059 | 0.0045 | 0.0074 | 0.0072 | 0.0026 | 0.0014 | 0.0019 |
|  | **Half-Life (d)** | 79.46 | 81.46 | 79.22 | 87.36 | 76.87 | 81.87 | 66.16 | 90.34 | 67.72 | 96.70 |
|  | **Half-Life Range Max** | 526.48 | 173.17 | 526.48 | 526.48 | 152.86 | 526.48 | 219.84 | 318.99 | 142.68 | 159.75 |
|  | **Half-Life Range Min** | 15.31 | 15.99 | 15.31 | 15.31 | 18.29 | 15.31 | 18.29 | 60.44 | 56.46 | 47.33 |
|  | **Relative Standard Error on Pop. Fit (%)** | 7.63 | - | - | - | - | - | - | - | - | - |
| **Anti-RBD IgA** | **Median Decay (d^-1^)** | 0.0152 | 0.0153 | 0.0152 | 0.0152 | 0.0152 | 0.0152 | 0.0151 | 0.0151 | 0.0153 | 0.0155 |
|  | **Decay IQR (d^-1^)** | 0.0009 | 0.0006 | 0.0009 | 0.0010 | 0.0007 | 0.0010 | 0.0008 | 0.0006 | 0.0005 | 0.0006 |
|  | **Half-Life (d)** | 45.58 | 45.38 | 45.60 | 45.57 | 45.64 | 45.55 | 45.83 | 45.84 | 45.36 | 44.84 |
|  | **Half-Life Range Max** | 54.80 | 53.36 | 54.80 | 54.80 | 53.36 | 53.56 | 54.80 | 51.94 | 46.59 | 46.77 |
|  | **Half-Life Range Min** | 33.86 | 39.59 | 33.86 | 33.86 | 38.69 | 34.33 | 33.86 | 40.87 | 43.73 | 40.55 |
|  | **Relative Standard Error on Pop. Fit (%)** | 2.68 | - | - | - | - | - | - | - | - | - |

**Table S8:** Serological neutralization of live ancestral SARS-CoV-2 *in vitro* following mRNA vaccination at 2-6 weeks and 6-months post-second dose of BNT162b2 or mRNA-1273.

|  | **Median Log(1/IC50) (IQR)** | | | | | | | | | |
| --- | --- | --- | --- | --- | --- | --- | --- | --- | --- | --- |
|  | **Entire Cohort** | **Sex** | | **Vaccine Type** | | **Immunological phenotype** | | | | |
|  |  | Male  (n = 17) | Female (n = 96) | BNT162b2 (n = 71) | mRNA-1273  (n = 42) | **Uninfected** | | HI  (n = 20) | Breakthrough (n = 8) | N+  PCR(-)  (n = 7) |
|  |  |  |  |  |  | CR  (n = 48) | NCR  (n = 30) |  |  |  |
| **Baseline**  **(n = 7)** | 0  (0) | - | - | - | - | - | - | - | - | - |
| **2-6 Weeks Post 2^nd^ Dose (n = 113)** | 2.45  (0.50) | 2.52  (0.59) | 2.45  (0.60) | 2.45  (0.61) | 2.51  (0.58) | 2.36  (0.43) | 2.17  (0.59) | 2.88  (0.82) | 2.60  (0.37) | 2.51  (0.13) |
| **6-months Post 2^nd^ Dose**  **(n = 113)** | 1.89  (0.60) | 1.90  (0.78) | 1.89  (0.60) | 1.62  (0.69) | 1.99  (0.46) | 1.69  (0.33) | 1.61  (0.62) | 2.20  (0.77) | 1.98  (0.37) | 2.05  (0.6) |

**Table S9:** A summary of decay rates in serological neutralizing capacity against ancestral SARS-CoV-2 in vaccinated LTCH staff from 2-6 weeks to 6-months post-second dose of BNT162b2 or mRNA-1273.

|  | **Entire Cohort**  **(n = 113)** | **Sex** | | **Vaccine Type** | | **Immunological phenotype** | | | | |
| --- | --- | --- | --- | --- | --- | --- | --- | --- | --- | --- |
|  |  | Male  (n = 17) | Female (n = 96) | BNT162b2 (n = 71) | mRNA-1273  (n = 42) | **Uninfected** | | HI  (n = 20) | Breakthrough (n = 8) | N+  PCR(-)  (n = 7) |
|  |  |  |  |  |  | CR  (n = 48) | NCR  (n = 30) |  |  |  |
| **Median Decay(d^-1^)** | 0.0017 | 0.0016 | 0.0017 | 0.0018 | 0.0016 | 0.0016 | 0.0017 | 0.0017 | 0.0018 | 0.0017 |
| **Decay IQR (d^-1^)** | 0.0011 | 0.0015 | 0.0010 | 0.0013 | 0.0007 | 0.0011 | 0.0013 | 0.0010 | 0.0007 | 0.0011 |
| **Half-Life (d)** | 408.43 | 427.80 | 408.14 | 391.21 | 440.02 | 429.19 | 413.79 | 410.09 | 388.40 | 399.49 |
| **Half-Life Range Min** | 1062.68 | 984.96 | 1062.7 | 1062.68 | 917.57 | 1062.68 | 917.57 | 984.96 | 554.65 | 803.63 |
| **Half-Life Range Max** | 91.07 | 107.01 | 91.07 | 91.07 | 108.64 | 91.07 | 108.64 | 106.76 | 222.06 | 176.99 |
| **Relative Standard Error on Pop. Fit (%)** | 7.00 | **-** | **-** | **-** | **-** | **-** | **-** | **-** | **-** | **-** |


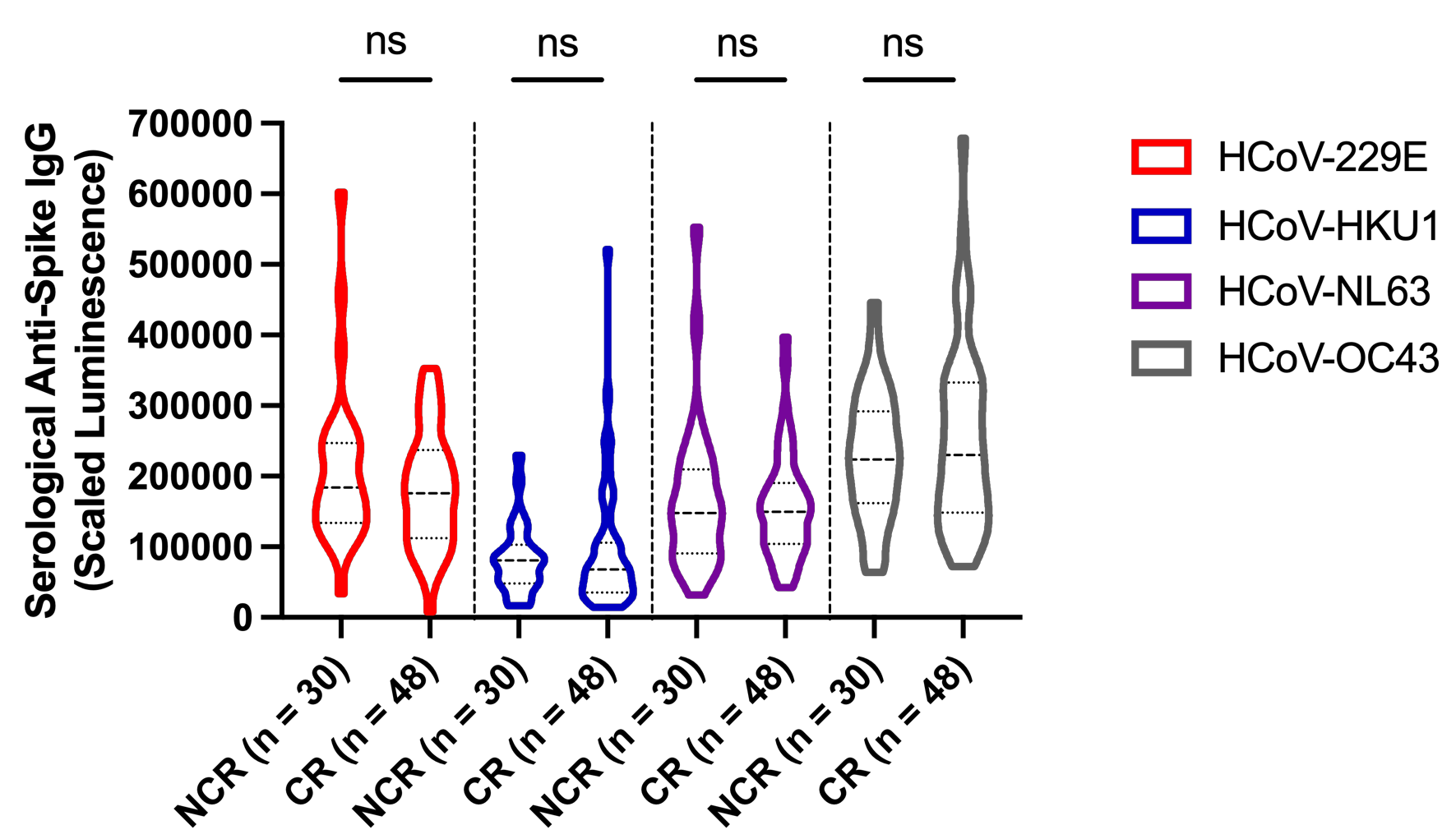


**Figure S1:** Comparisons of serological anti-spike IgG antibody levels against HCoV-229E (red), HCoV-HKU1 (blue), HCoV-NL63 (purple), and HCoV-OC43 (grey) between non-cross-reactive and cross-reactive mRNA vaccinees at 2-6 weeks post-second dose.


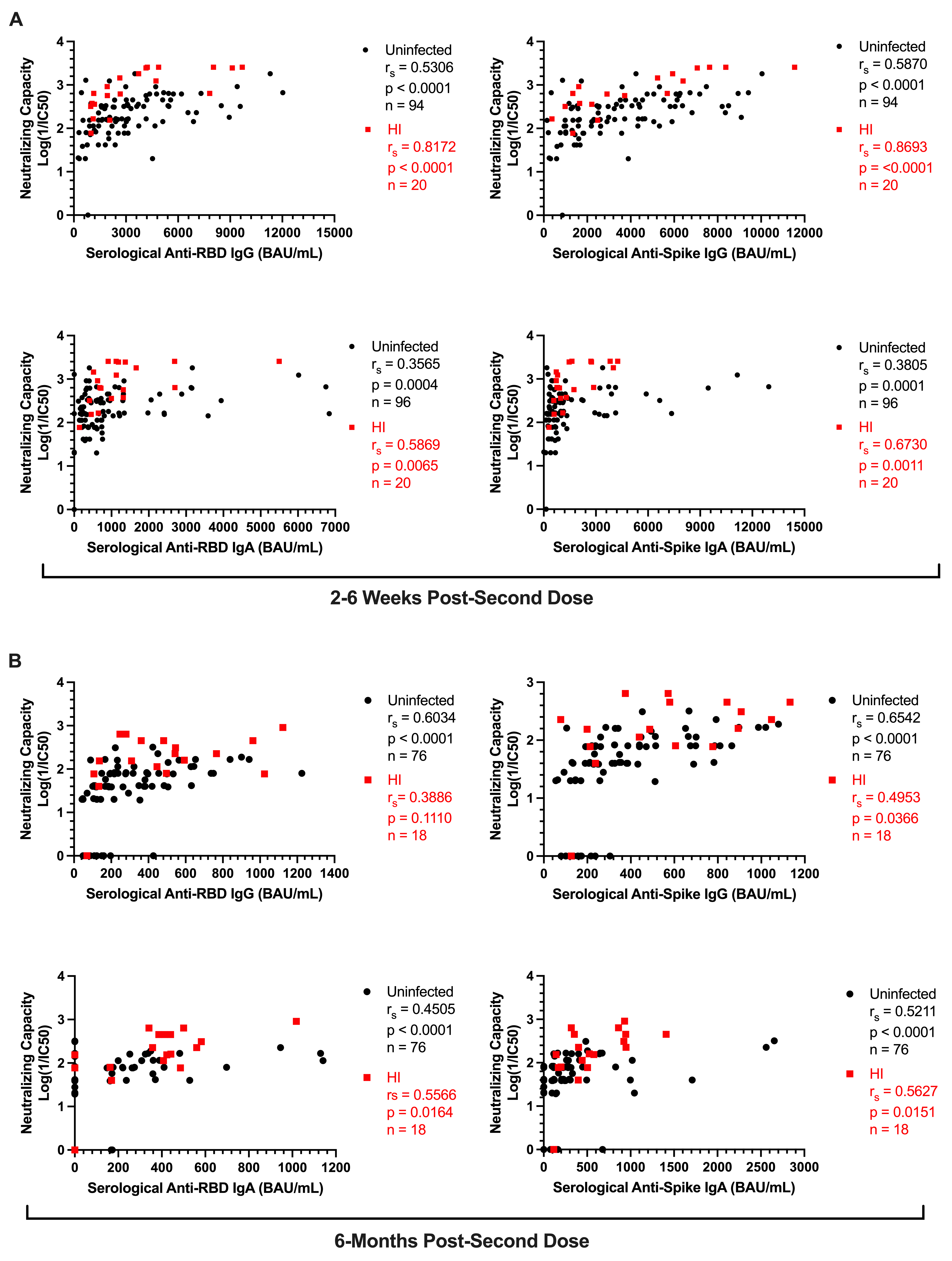


**Figure S2:** Correlations observed between serological anti-spike/RBD IgG and IgA antibody levels and neutralizing capacity against live ancestral SARS-CoV-2 in uninfected and HI vaccine recipients at (A) 2-6 weeks and (B) 6-months post-second dose of BNT162b2 or mRNA-1273. Black circles = uninfected; red squares = HI.

**

**

**Figure S3:** A summary of spike-specific (black) and non-spike-specific (red) T-cell responses in NCR vaccinees at 2-6 weeks and 6-months post-second dose. Non-spike-specific T-cell responses are cumulative of N-, E-, M-, and NSP-specific responses.

1. 0 = Asymptomatic; 1 = Mild symptoms, no fever; 2 = Mild symptoms with fever, not hospitalized [↑](#footnote-ref-1)
2. Exhibited an asymptomatic PCR-confirmed breakthrough infection 14 days following second vaccine dose, but 11 days prior to sample acquisition [↑](#footnote-ref-2)
